# Detecting prognostic biomarkers of breast cancer by regularized Cox proportional hazards models

**DOI:** 10.1186/s12967-021-03180-y

**Published:** 2021-12-20

**Authors:** Lingyu Li, Zhi-Ping Liu

**Affiliations:** Department of Biomedical Engineering, School of Control Science and Engineering, Shandong University, Jinan, 250061 China

**Keywords:** Breast cancer, Regularized Cox proportional hazards model, Feature selection, Biomarker, Prognostic risk score

## Abstract

**Background:**

The successful identification of breast cancer (BRCA) prognostic biomarkers is essential for the strategic interference of BRCA patients. Recently, various methods have been proposed for exploring a small prognostic gene set that can distinguish the high-risk group from the low-risk group.

**Methods:**

Regularized Cox proportional hazards (RCPH) models were proposed to discover prognostic biomarkers of BRCA from gene expression data. Firstly, the maximum connected network with 1142 genes by mapping 956 differentially expressed genes (DEGs) and 677 previously BRCA-related genes into the gene regulatory network (GRN) was constructed. Then, the 72 union genes of the four feature gene sets identified by Lasso-RCPH, Enet-RCPH, $L_{0}$-RCPH and SCAD-RCPH models were recognized as the robust prognostic biomarkers. These biomarkers were validated by literature checks, BRCA-specific GRN and functional enrichment analysis. Finally, an index of prognostic risk score (PRS) for BRCA was established based on univariate and multivariate Cox regression analysis. Survival analysis was performed to investigate the PRS on 1080 BRCA patients from the internal validation. Particularly, the nomogram was constructed to express the relationship between PRS and other clinical information on the discovery dataset. The PRS was also verified on 1848 BRCA patients of ten external validation datasets or collected cohorts.

**Results:**

The nomogram highlighted that the importance of PRS in guiding significance for the prognosis of BRCA patients. In addition, the PRS of 301 normal samples and 306 tumor samples from five independent datasets showed that it is significantly higher in tumors than in normal tissues ($P<0.05$). The protein expression profiles of the three genes, i.e., *ADRB1*, *SAV1* and *TSPAN14*, involved in the PRS model demonstrated that the latter two genes are more strongly stained in tumor specimens. More importantly, external validation illustrated that the high-risk group has worse survival than the low-risk group ($P<0.05$) in both internal and external validations.

**Conclusions:**

The proposed pipelines of detecting and validating prognostic biomarker genes for BRCA are effective and efficient. Moreover, the proposed PRS is very promising as an important indicator for judging the prognosis of BRCA patients.

**Supplementary information:**

**Supplementary information** accompanies this paper at 10.1186/s12967-021-03180-y.

## Background

Breast cancer (BRCA) is the second leading cause of cancer mortality in women [1], and its incidence increases with age [2]. At present, the incidence of BRCA remains high and lesions may occur if treatment is not timely or improperly treated [3]. To date, relatively few reliable prognostic biomarkers of BRCA have been identified. It is of great significance to study biologically interpretable BRCA prognostic biomarkers. With the development of high-throughput technology, gene expression profiling can measure the expression levels of thousands of genes in a parallel manner and has been widely used to discover new biomarkers [4]. It has become increasingly obvious that more and more attention needs to pay to the relationship between gene expression profiles and survival phenotypes, such as the time of cancer recurrence or death [5]. In addition, identifying the prognostic genes related to BRCA survival can provide new information for clinical decision-making, diagnosis, prognosis, and treatment options of BRCA patients [6].

Numerous studies have identified some prognostic biomarkers for BRCA using high-throughput screening methods, which indicates the prognosis biomarkers for predicting patients are very promising. For instance, Kim et al. [7] applied a network-regularized Cox regression model to perform feature selection on mRNA expression and clinical data, and developed a new BRCA prognostic score system. Yan et al. [8] employed the Cox regression to mine DNA methylation data and identified seven BRCA-related DNA methylation signatures. Zhang et al. [9] combined multivariate Cox analysis and artificial-intelligence-based algorithms to select 17 immune genes as potential prognostic biomarkers for BRCA. Sarkar et al. [10] implemented an ensemble of feature selection methods to identify 27 miRNAs as biomarkers that are highly correlated with multiple BRCA subtypes. Li et al. [11] proposed the scPrognosis method using single-cell RNA sequencing (scRNA-seq) data to improve the prognosis of BRCA and successfully identified ten BRCA biomarkers. However, most of these studies only select differentially expressed genes (DEGs), and only perform co-expression analysis by using univariate Cox analysis to reduce the dimensionality of variables. They are also very limited in the exploration of possible biomarkers and their prognostic value.

So far, it might not be enough to screen prognosis biomarkers of BRCA purely using a single dataset or one kind of information [8]. The complexity of genomic data and prior knowledge promote us to integrate the aforementioned BRCA-related data and information for accurate biomarker discovery. Namely, MammaPrint successfully selects BRCA signatures that are differentially expressed in two different sets of BRCA tumors [12]. The online consensus survival analysis web server for breast cancers (OSbrca) collected the prognostic ability of 128 previously published BRCA biomarkers [13]. Moreover, gene ontology (GO) describes the dysfunctions of genes that provide more candidates related to the prognosis of BRCA [14]. KEGG is an integrated database that covers a variety of knowledge including BRCA-related genes in the form of a pathway [15]. All of them will benefit us discover important genes related to cancer dysfunctions and select more reliable and biologically interpretable biomarkers [4].

In survival analysis, the response variable is the time at which the event of interest such as ‘death’ occurred. The main goal is to identify covariates that increase the risk of the event of interest [16]. The semi-parametric Cox proportional hazard (CPH) model originally proposed by Cox [17] uses the partial likelihood structure under the proportional hazard assumption to estimate the regression coefficients, avoiding the selection of specific parameter distribution for survival time [18]. Recently, more and more studies have been focusing on analyzing the data that contains high-dimensional variables. In fact, only some of them are generically related to the response variable. How to select significant variables effectively contributing to the result is an important but not always easy task [16]. In addition to the high dimensionality, the expression profiles of some genes are often highly correlated, which creates the problem of high collinearity [19]. To meet these two problems, the most commonly used method is to use penalized partial likelihood, i.e., regularization [5]. The regularized regression model with penalty provides an attractive method to build predictive model from high-dimensional data, which is an embedded machine learning procedure that can simultaneously select the features and fit the model [20].

Tibshirani [21] first extended Lasso to generalized linear regression models and time-to-event endpoints. The CPH model with Lasso penalty shows good feature selection effects. Later, Li and Luan [22] proposed the CPH model with ridge penalty and clarified the limitation of using all genes for prediction, but it does not provide a method for selecting feature genes for prediction. In addition to the well-known $L_1$ norm (Lasso) and $L_2$ norm (Ridge), convex penalty functions such as the linear combination of $L_1$ and $L_2$ norm (i.e., Elastic net, abbreviated as Enet) have also been proposed for feature selection as well as model prediction [23]. Next, various other non-convex penalty functions, e.g., $L_{1/2}$ (the regularized representative of $L_q (0<q <1)$) [5], $L_0$ [24], SCAD [25] and MCP [26], have good performance in sparse optimization. Some of the penalties have been proven to have fantastic properties, such as unbiasedness and oracle property in variable selection [5, 27]. Obviously, discovering diagnostic or prognostic biomarkers is equivalent to select features from high-dimensional variables. In biomedicine, a central topic of cancer genomics is to identify interpretable biomarkers for better disease prognosis [28].

In this paper, we aim to develop a computational method for prognostic biomarker discovery in BRCA by the regularized Cox proportional hazard (RCPH) models from gene expression profiling data. Firstly, we identify DEGs across breast tumors and control samples from the publicly available RNA sequencing (RNA-seq) data. Combining DEGs with BRCA-related genes from the documented databases of prior knowledge and mapping them into an integrative GRN to extract the maximum connected component, we feed these genes included in connected network into the RCPH model with the seven penalized $L_q$ functions for selecting features and recognize that four gene subsets obtained by the Lasso, Enet, $L_0$ and SCAD penalties relatively achieve larger C-index and smaller *P*-value on the internal validation data. Secondly, we take the union of 72 genes from the four optimal feature subsets as prognostic biomarkers of BRCA and validate the identified signatures from various aspects. By extracting the network component from a comprehensive GRN [29], we construct the BRCA-specific network structure in these prognostic biomarker genes. Subsequently, the enriched function terms imply 72 biomarkers are significantly related to BRCA (*P*=1.6e−11). The results from literature validations indicate 51 of the biomarkers have been confirmed to be related to BRCA. Thirdly, we establish a prognostic risk score (PRS) system by univariate and multivariate Cox regression analysis. We perform the survival analysis to investigate the PRS values in the 1080 patients on the internal validation dataset. In particular, we also construct a nomogram to explore the influence of PRS and other clinical factors on the survival probability of BRCA patients on the biomarker discovery data. Finally, we evaluate the PRS values of a total of 1848 patients from ten external verification datasets. We also calculate the PRS of 301 controls and 306 tumor samples from five independent datasets to show their distinctiveness. The protein expression profiles are further used to validate the difference of the three prognostic genes involved in the PRS system between breast tumors and normal tissues. The source code and data used in this paper can be found at https://github.com/zpliulab/CoxReg.

## Methods

### Data

The gene expression profiling data for BRCA patients and their corresponding clinical details are downloaded from The Cancer Genome Atlas (TCGA) database (https://cancergenome.nih.gov/) and Gene Expression Omnibus (GEO) database (https://www.ncbi.nlm.nih.gov/geo/). Table 1 summarizes the basic sample information in the biomarker discovery dataset (TCGA, 1080 patients) and the external validation datasets (GSEGSE1456, GSE2034, GSE7390, GSE17705, GSE21653 and GSE35629, totally 1848 patients) respectively.

**Table 1:** The basic information and clinical characteristics of patients with BRCA patients

Characteristics		Datasets						
		Discovery	External validation (totally 1848 patients)					
Cohort		TCGA	GSE1456	GSE2034	GSE7390	GSE17705	GSE21653	GSE35629
Platform		DCC	GPL96	GPL96	GPL96	GPL570	GPL570	GPL1390
Survival		OS	OS, DMFS	DMFS	OS, DFS, TDM	RFS	DFS	OS, RFS
# of samples		1080	159, 159	286	198, 198, 198	298	248	53, 51
# of genes		20220	13701	13701	13701	13701	21835	7800
Age	$\le 60$	587			195		157	
	$>60$	479			3		91	
	NA	14			0		0	
	Average	58			46		55	
	IQR	(49, 67)			(42, 51)		(45, 66)	
Tumor size (mm)					21.81			
Stage	I	181	28		30		43	
	II	611	58		83		84	
	III	246	61		83		121	
	IV	20			0		0	
	V	14			0		0	
	NA	8	12		2		4	
T stage	T1	279					57	
	T2	626					121	
	T3	134					63	
	T4	38					0	
	NX	3					7	
N stage	N0	505						
	N1	359						
	N2	120						
	N3	76						
	NX	20						
M stage	M0	896						
	M1	22						
	MX	162						
Status	0	928	130, 119	276	142, 107, 147	227	169	29, 30
	1	152	29, 40	10	56, 91, 51	71	79	24, 21
References		[61]	[62]	[63]	[64]	[65]	[66]	[67]

*IQR: Interquartile range (1, 3).* OS* overall survival,* DMFS* distant metastasis-free survival,* TDM* time to distant metastasis,* DFS* disease-free survival,* RFS* relapse free survival. For OS: 1 = dead from BRCA, 0 = alive or censored. For DMFS: 1 = relapse, 0 = no relapse or censored. For TDM, DFS and RFS: 1 = event, 0 = censoring

From TCGA, we collect 1093 samples with 20501 genes individually. The counts of gene expression *x* are transformed to normalized value by $\log (x+1)$. After data preprocessing, 20220 genes have remained for subsequent research. We get the survival information for each sample and discard individuals with survival time less or equal to 0. As a result, 1080 samples are left. For the microarray data from GEO, we download their series matrix files. Some important clinical characteristics including age, tumor size, pathologic stage (I, II, III, IV, V and NA), pathology stage (T, N, M) and survival status are available. The datasets listed in Table 1 are used to discover and verify prognostic biomarkers of BRCA.

Moreover, we also use the RNA-seq data of TCGA and the microarray data of GEO (GSE5764, GSE7904, GSE10780 and GSE42568), a total of 607 patients (301 normals and 306 tumors), as the external validation datasets to verify the stability and robustness of the PRS index for prediction in BRCA. The details are listed in Table 2.

**Table 2:** The details of the datasets for prognostic prediction of PRS

Datasets	Platforms	# of samples	# of genes	References
TCGA	DCC	224 (112 Normal / 112 Tumor)	20222	[61]
GSE5764	GPL570	15 (10 Normal / 5 Tumor)	21835	[68]
GSE7904	GPL570	62 (19 Normal / 43 Tumor)	16452	[69]
GSE10780	GPL570	185 (143 Normal / 52 IDC)	21835	[70]
GSE42568	GPL570	121 (17 Normal / 104 IDC)	21835	[71]

*IDC Invasive ductal carcinomas. Histopathological BRCA subtypes: invasive ductal (IDC), invasive lobular (ILC), mixed ductal/lobular (Mixed), and other-type (Other) carcinoma [72]

### Differential gene identification and prior information integration

We select DEGs simultaneously identified by two approaches. Firstly, we select the samples with both tumor tissues and its adjacent normal tissues from TCGA. Then we identify the DEGs across the 224 samples (112 normals and 112 tumors) by DEseq2 [30], which results in 489 genes with adjusted *P*-value (*P*.adj) $< 0.01$ and $|\log$(FC)$| > 3.322$. Secondly, the gene expression data of 1080 samples (928 alive and 152 dead) are also screened by DEseq2 and finally 501 genes with *P*.adj $< 0.01$ are also regarded as DEGs. Thus we totally identify 956 union DEGs which are listed in Additional file 1: Table S1.

As known, prior knowledge about BRCA is very important in identifying potential prognostic biomarkers. Here, we integrate the interesting genes from five kinds of prior knowledge, namely, the 147 genes in breast pathway from KEGG [15], the 519 genes related to BRCA from the top-ranked GO terms sorted in gene ontology annotations (GOA) [14], the known MammaPrint BRCA signatures with 70 genes [12], the OSbrca webserver about BRCA diagnosis with 128 genes [13] and the 10 BRCA prognosis signatures selected by scPrognosis from scRNA-seq data [11]. Furthermore, we map the total of 1633 genes into an integrated human gene regulatory network documented in RegNetwork [29] to extract the maximum connected component. Consequently, 1142 genes linked as a network are retained for screening prognostic biomarkers using the proposed RCPH models.

### Framework

**Fig. 1:**
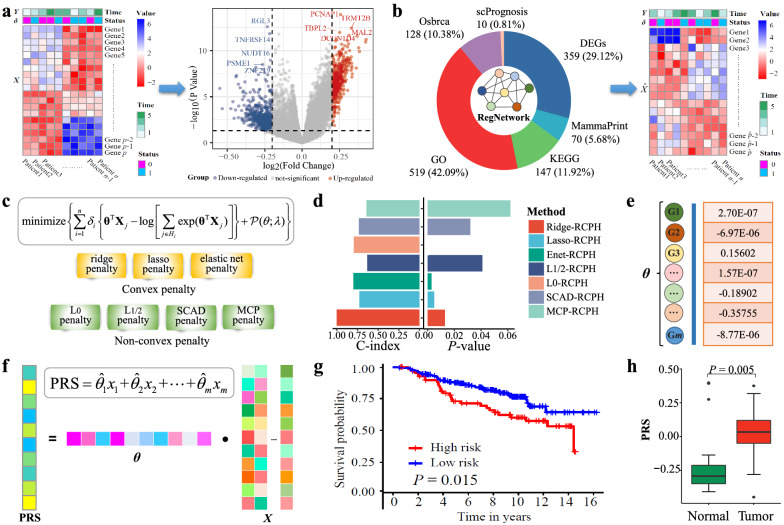
The framework of detecting and verifying prognostic biomarkers of BRCA from gene expression data by the RCPH methods. **a** Download the publically available RNA-seq data and then select DEGs. **b** Add the prior knowledge from KEGG, GO, MammaPrint, OSbrca, and scPrognosis to DEGs and integrate all of them with RegNetwork to obtain a connected network component, and extract the gene expression values accordingly. **c** Apply the RCPH models on the network-structured data to select the feature genes of BRCA. **d** Choose the optimal feature subsets via the assessment of C-index and P-value. **e** Identify the genes with non-zero regression coefficients as the potential BRCA biomarkers. **f** Establish the PRS model based on statistically significant genes from the Cox model in order to make response predictions for prognosis and treatment of BRCA. **g** Perform survival analysis using the PRS index to investigate its prognostic performance. **h** Explore the significance and differences of PRS index in normal and tumor tissues

Figure 1 illustrates the framework of detecting and verifying biomarkers and PRS index for the prognosis prediction of BRCA from high-throughput transcriptomics data by the RCPH models. As shown in Fig. 1, we firstly download the RNA-seq data from TCGA and pick out DEGs. Secondly, we combine the DEGs with the candidate genes selected from KEGG, GO, MammaPrint, OSbrca, and scPrognosis. In order to enhance the connection strengths between genes and make the biomarkers more biologically meaningful, we link the genes in the candidate set with the regulations documented in RegNetwork [29]. We thus obtain 1142 genes connected in the form of a network. Thirdly, we apply the RCPH models to select genes with non-zero regression coefficients to obtain seven feature subsets on the training dataset ($70\%$). We also evaluate the survival analysis performances of the seven RCPH methods on the testing dataset ($30\%$) via the assessment of C-index and *P*-value. Fourthly, to select more robust biomarkers, the 72 union feature genes selected by four RCPH models with Lasso, Enet, $L_0$ and SCAD penalty (C-index $\ge 0.700$ and *P*-value $\le 0.050$) are identified as the prognostic biomarkers of BRCA. Fifthly, we construct a PRS system based on univariate and multivariate Cox regression and then perform survival analysis to investigate its prognostic performance on 1080 patients in the internal validation and 1848 patients in the external validation. Sixthly, we compare the PRS values for checking their differences in normal and tumor tissues in the independent external validation datasets.

### Regularized Cox proportional hazards (RCPH) models

For convenience, we introduce some notations here. Assuming that *T* represents the potential survival time for each patient, we observe the quantity $Y = \min (T, C)$, where *C* is the censoring time [19]. In order to explore the relationship between a patient’s survival time and the corresponding gene expression levels $\varvec{X}_1, \varvec{X}_2, \cdots , \varvec{X}_n$, we suppose the dataset $\mathscr {D}$ has *n* samples with the form

1 $$\begin{aligned} \mathscr {D} = \{ (\varvec{X}_1, y_1, \delta _1), (\varvec{X}_2, y_2, \delta _2), \cdots , (\varvec{X}_n, y_n, \delta _n)\}, \end{aligned}$$

where $\varvec{X}_i =(x_{i1}, x_{i2}, \cdots , x_{ip})^{\mathrm{{T}}} \in {\mathbb {R}}^{p}$ represents the *p*-dimension covariates with $i = 1,2, \cdots , n$, and

$$\begin{aligned} \delta _i = \left\{ \begin{array}{*{35}{l}} 1, &{} \text {right censoring time}, \\ 0, &{} \text {no censoring}, \\ \end{array} \right. \end{aligned}$$

is a binary censoring indicator variable representing whether $y_i$ is dead time or censoring time.

To study the hazard of disease recurrence or death at time *t*, the following CPH model is prosed by [17]

2 $$\begin{aligned} h(t,\varvec{X}_i)&= h_0(t) \cdot \exp ( \theta _1 x_{i1} + \theta _2 x_{i2} + \cdots +\theta _p x_{ip} ) \nonumber \\&= h_0(t) \cdot \exp (\varvec{X}_i^{\mathrm{{T}}} \varvec{\theta }), \end{aligned}$$

where $h(t,\varvec{X}_i)$ is the hazard for the *i*-th sample at time *t*, $h_0(t)$ is the baseline hazard, i.e., the hazard of each sample at $\varvec{X}_i = 0$, and $\varvec{\theta } = (\theta _1, \theta _2, \cdots , \theta _p)^{\mathrm{{T}}} \in {\mathbb {R}}^{p}$ is the unknown *p*-dimensional regression coefficients of the *i*-th sample to be solved [31].

By a simple calculation for Eq.(2), its partial likelihood function is

3 $$\begin{aligned} \mathscr {L}(\varvec{\theta } |\mathscr {D}) = \prod _{i|\delta _i = 1}^{n} \frac{\exp \{\varvec{X}_i^{\mathrm{{T}}} \varvec{\theta }\}}{\sum _{j \in H_{i}} \exp \{\varvec{X}_j^{\mathrm{{T}}} \varvec{\theta }\}}, \end{aligned}$$

where $t_i$ is the survival time (observed or censored) of the *i*-th sample [32], $H_{i}=\{ j \ |\ t_j > t_i,\ j = 1,2, \cdots , n\}$ is the risk set of alive samples at time $t_i$ [22, 33].

To estimate the regression coefficients $\varvec{\theta }$ from Eq.(3), we need to maximize its log partial likelihood function

4 $$\begin{aligned} \mathscr {L}(\varvec{\theta } |\mathscr {D})&= \log \mathscr {L(\varvec{\theta } |\mathscr {D})} \nonumber \\&= \sum _{\left\{ i \mid \delta _{i}=1\right\} }^{n} \log \left[ \frac{\exp \{\varvec{X}_i^{\mathrm{{T}}} \varvec{\theta }\}}{\sum _{j \in H_{i}}^{n} \exp \{\varvec{X}_j^{\mathrm{{T}}} \varvec{\theta } \}} \right] \nonumber \\&= \sum _{i=1}^{n} \delta _i \left\{ \varvec{X}_i^{\mathrm{{T}}} \varvec{\theta } - \log \left[ \sum _{j \in H_{i}} \exp \{\varvec{X}_j^{\mathrm{{T}}} \varvec{\theta }\} \right] \right\} . \end{aligned}$$

However, in practice, the Eq.(4) cannot be directly used to estimate the coefficients $\varvec{\theta }$, and it is expected that not all these *p* genes are contributed to predict the survival outcomes. Especially in the case of high-dimensional small sample data (e.g., microarray data and RNA-seq data) where the dimensionality *p* is usually much larger than the sample size *n*, i.e., $p>> n$ [5]. That is to say, some components $\theta _k\ (k = 1, 2, \cdots , p)$ of coefficient vector $\varvec{\theta }$ are zeros under the real circumstances. Therefore, the regularization methods, such as *Lq* penalty functions $\mathscr {P}(\varvec{\theta }; \lambda )$ shown in Table 3, are proposed to solve Eq.(4) [34]. Additionally, the properties (e.g., unbiased, sparse, continuous, convex and oracle property) of $\mathscr {P}(\varvec{\theta }; \lambda )$ have been discussed in [27].

**Table 3:** ${L_q}$ penalty functions of regularization term used in RCPH models

Methods	Formulas	References
Ridge	$\mathscr {P}(\varvec{\theta };\lambda ) = \lambda \sum \limits _{j=1}^{p} \theta _{j} ^{2}$	[73]
Lasso	$\mathscr {P}(\varvec{\theta };\lambda ) = \lambda \sum \limits _{j=1}^{p}|\theta _{j}|$	[74]
Enet	$\mathscr {P}(\varvec{\theta }; \lambda ) = \lambda \Big [\alpha \sum \limits _{j=1}^{p} | \theta _{j}|+ (1-\alpha )\sum \limits _{j=1}^{p}\theta _{j}^{2}\Big ]$	[75]
$L_{0}$	$\mathscr {P}(\varvec{\theta };\lambda ) = \lambda \sum \limits _{j=1}^{p}{1}\left[ {{\theta }_{j}}\ne 0 \right]$	[76]
$L_{1/2}$	$\mathscr {P}(\varvec{\theta };\lambda ) = \lambda \sum \limits _{j=1}^{p}|\theta _{j}|^{\frac{1}{2}}$	[77]
SCAD	$\mathscr {P}(\varvec{\theta };\lambda )=\sum \limits _{j=1}^{p}\mathscr {P}_{a}\left( |\theta _{j}|;\ \lambda \right)$,	[78]
	where $$\mathscr {P}_{a}(|\theta |;\lambda )=\left\{ \begin{array}{*{35}{l}} \lambda |\theta |, &{} |\theta |\le \lambda , \\ \frac{-\left( \theta ^{2} -2a\lambda |\theta |+ \lambda ^{2} \right) }{2(a-1)}, &{} \lambda < |\theta | \le a\lambda , \\ \frac{(a+1) \lambda ^2}{2}, &{} |\theta |>a\lambda . \\ \end{array} \right.$$	
MCP	$\mathscr {P}(\varvec{\theta };\lambda )=\sum \limits _{j=1}^{p}\mathscr {P}_{a}\left( \theta _{j};\ \lambda \right)$,	[79]
	where $$\mathscr {P}_{a}(\theta ;\lambda ) = \left\{ \begin{array}{*{35}{l}} \lambda |\theta |-\frac{\theta ^2}{2a}, &{} |\theta |\le \lambda a, \\ \frac{\lambda ^2 a}{2}, &{} |\theta |>\lambda a. \\ \end{array} \right.$$	

Adding the regularization term $\mathscr {P}(\varvec{\theta }; \lambda )$ to the negative of Eq.(4) and minimizing the sum of them, then the regularized Cox proportional hazards model (RCPH) can be obtained

5 $$\begin{aligned} \varvec{\theta } =\arg \min \left\{ -\mathscr {L}(\varvec{\theta }|\mathscr {D}) + {\mathscr {P}} \left( \varvec{\theta };{\lambda }\right) \right\} , \end{aligned}$$

where $\lambda$ is a positive tuning parameter used to balance the loss function $-\mathscr {L}(\varvec{\theta }|\mathscr {D})$ and penalty function ${\mathscr {P}} \left( \varvec{\theta };{\lambda }\right)$. The RCPH model (5) selects important features and estimates the error of model simultaneously by shrinking some components of regression coefficient $\varvec{\theta }$ to zeros.

### Turning parameters optimization

For the RCPH model, the tuning parameter $\lambda$ is determined by *K*-fold cross-validation (CV) [35]. The discovery dataset is split into *K* folds, where $K -1$ folds of data are used to train the model and the left-out fold data is used for validation. The procedure is performed *K* times for each parameter $\lambda$. Then, the test data itself will choose the parameter with the best goodness of fit for the training data and with the best performance to the new data.

The optimal value $\lambda$ is estimated by minimizing the cross-validation log partial likelihood (CV-LPL) [32], i.e., the sum of the contributions of each sample to the log partial likelihood, which has been demonstrated to perform well in the context of the RCPH models [36]. The CV-LPL is defined as

6 $$\begin{aligned} \text {CV-LPL}(\lambda )=-\sum _{k=1}^{K}\left\{ \mathscr {L}\left( \hat{\varvec{\theta }}^{(-k)}(\lambda )\right) -\mathscr {L}^{(-k)}\left( \hat{\varvec{\theta }}^{(-k)}(\lambda )\right) \right\} , \end{aligned}$$

where $\hat{\varvec{\theta }}^{(-k)}(\cdot )$ represents the estimating value of $\varvec{\theta }$, it is obtained when the *k*-th fold of the data is left out with a given $\lambda$ for the model, $\mathscr {L}(\cdot )$ is the log partial likelihood using all *n* samples, while $\mathscr {L}^{(-k)}(\cdot )$ is the log partial likelihood excluding the *k*-th fold samples [4]. Note that the choice of *K* is often depend on the size of the dataset. When *K* is given, we can compute the coefficients $\hat{\varvec{\theta }}^{(-k)}(\lambda )$ accordingly [19].

### Performance evaluation

#### Prognostic risk score (PRS) construction

To establish a PRS system for prognosis and treatment response prediction of BRCA, we propose the following model

7 $$\begin{aligned} \text {PRS} = \hat{\theta _1} x_1 + \hat{\theta _2} x_2 + \cdots + \hat{\theta _m} x_m, \end{aligned}$$

where *m* is the total number of independent prognostic genes, $x_i$ represents the expression value of *i*-th gene, $\hat{\theta _i}$ represents the regression coefficient of gene *i* derived from the multivariate Cox regression model. The optimal cut-off value is automatically generated by X-Tile [37]. Subsequently, all patients could be divided into a high-risk group and a low-risk group based on the optimal cut-off value of PRS.

#### Concordance index (C-index)

Here we employ concordance index (C-index) [11] to measure the discrimination of predict value and true value to validate the prediction ability of the RCPH models in BRCA prognosis. For the right censoring data, C-index can be defined as

8 $$\begin{aligned} \text {C-index} = \frac{\sum _{i} \sum _{j} \mathscr {I}\left( f_{i}<f_{j} \wedge \delta _{i}=1\right) }{\sum _{i} \sum _{j} \mathscr {I}\left( t_{i}<t_{j} \wedge \delta _{i}=1\right) }, \end{aligned}$$

where $\mathscr {I}(\star )$ is an indication function, if $\star$ is true, then $\mathscr {I}(\star )=1$, otherwise $\mathscr {I}(\star )=0$. $f(\cdot )$ is the survival function, when $f_i >f_j$ and $\delta _j=1$ it holds $t_i>t_j$. In particular, Eq.(8) is a fraction, ranges from 0.5 to 1, of all pairs of samples which predicted survival times are correctly ordered among all samples that can actually be ordered. The bigger the C-index is, the more accurate of a model will be [11]. $\text {C-index}=0.5$ indicates that the model has no predictive effect, while $\text {C-index}=1$ indicates that the prediction results are completely consistent with reality.

#### Kaplan-Meier curve

Survival curves are estimated by the Kaplan-Meier (KM) estimator [38] combining with the two-sided Log-rank test to identify whether the high-risk and low-risk groups exist a statistically significant difference in survival patterns. The KM method is a non-parametric method of estimating survival probability from observed survival time. For a good prediction model, the KM curves should not overlap with different groups. If $P \le 0.05$ in the Log-rank test, it implies that the difference of survival curves is statistically significant.

#### Nomogram building

To express the relationship between variables in the predictive model, the six clinical indexes, namely PRS, years to birth (i.e., age), the pathologic stage (i.e., tumor grade), pathology T stage, pathology N stage, and pathology M stage, are incorporated to construct a nomogram for the survival probability prediction of the OS at 1-, 3- and 5-years for BRCA patients [8]. Simultaneously, the calibration curves for predicting 1-, 3- and 5-years are plotted to predict the effectiveness of the nomogram. The higher the coincidence degree of the fitting line (red line) and the diagonal line (blue dot), the better performance the nomogram exhibits.

#### Functional enrichment analysis

To illustrate the enriched functions underlying these identified prognostic biomarkers, we firstly employ the GO [14] functional enrichment analysis via clusterprofiler [39] and the pathway enrichment analysis on Metascape [40] (http://metascape.org/). Furthermore, we build up a semantic similarity measure (SS-measure) to access the enriched functions with the known cancer hallmarks [41]. The GO terms of cancer hallmarks are available from Carbon et al. [14], and we confirm the quality of GO semantic similarity between these enriched terms with the hallmark terms by the following method.

Suppose there are two GO term sets: $\mathscr {G}_1=\{ go_{11}, go_{12}, \cdots , go_{1k} \}$ and $\mathscr {G}_2=\{go_{21}, go_{22}, \cdots , go_{2l} \}$, let the similarity of each node $go_{1i}\ (i = 1,2, \cdots , k)$ belonging to set $\mathscr {G}_1$ and set $\mathscr {G}_2$ be the maximum value of the similarity of each GO term in set $\mathscr {G}_1$ and set $\mathscr {G}_2$, then it holds that

9 $$\begin{aligned} {\text {Sim}}(GO_{1i}, \mathscr {G}_{2}) = \max _{1 \le j \le l}\left\{ S_{\mathscr {G}_{2}}(Go_{1i}, GO_{2j})\right\} . \end{aligned}$$

Correspondingly, the similarity between each term $GO_{2j}\ (j = 1,2, \cdots , l)$ in set $\mathscr {G}_2$ and set $\mathscr {G}_1$ is

10 $$\begin{aligned} {\text {Sim}}(GO_{2j}, \mathscr {G}_{1}) = \max _{1 \le i \le k}\left\{ S_{\mathscr {G}_{1}}(GO_{2j}, GO_{1i})\right\} . \end{aligned}$$

Therefore, the SS-measure of two sets of GO terms is defined as the arithmetic average of the above two similarity indicators [42], i.e.,

11 $$\begin{aligned} {\text {SS-measure}}(\mathscr {G}_{1}, \mathscr {G}_{2}) = \frac{\max \limits _{1 \le i \le k} {\text {Sim}}(GO_{1i}, \mathscr {G}_{2}) + \max \limits _{1 \le j \le l} {\text {Sim}}(GO_{2j}, \mathscr {G}_{1}) }{k+l}, \end{aligned}$$

where $S_{\mathscr {G}}$ is defined by Eq.(3) in Wang et al. [43].

## Results

### Selected feature genes as biomarkers

We start from the 1142 genes contained in RegNetwork [29] in the discovery dataset (TCGA). Without loss of generality, we randomly divide the discovery dataset into a training dataset and a testing dataset by 7:3 of all samples. We implement the RCPH models according to the RNA-seq data and clinical information of samples on the training dataset. For each RCPH model, we randomly divide the above training and testing datasets 20 times of experiment. It is extremely necessary to take into account the robustness of feature selection during the training process instead of directly using the once result. Consequently, the union genes corresponding to non-zero coefficients in the 20 experiments are regarded as the selected feature subset.

As shown in Table 4, the average values of C-index and *P*-value obtained in 20 experiments on the testing dataset are used as the final C-index and *P*-value. In each experiment, we use 10-fold CV-LPL on the same training dataset to get the optimal tuning parameters for each RCPH model and validate on the testing dataset respectively.

**Table 4:** The results of feature selection on the discovery dataset performed by seven different RCPH models

Methods	Training dataset ($70 \%$)	Testing dataset ($30 \%$)	
	# of features	C-index ± Std. Dev	P-value ± Std. Dev
Ridge-RCPH	1142	$1.000 \pm 0.000$	$0.013 \pm 0.001$
Lasso-RCPH	47	$0.726 \pm 0.022$	$0.005 \pm 0.007$
Enet-RCPH	66	$0.798 \pm 0.044$	$0.003 \pm 0.002$
$L_{1/2}$-RCPH	4	$0.629 \pm 0.000$	$0.041 \pm 0.000$
$L_{0}$-RCPH	17	$0.794 \pm 0.000$	$0.000 \pm 0.000$
SCAD-RCPH	42	$0.731 \pm 0.046$	$0.032 \pm 0.018$
MCP-RCPH	22	$0.639 \pm 0.019$	$0.062 \pm 0.044$

*Std. Dev Standard deviation

The performance of these RCPH models in feature selection is important to determine the final results of biomarker discovery. Here the RCPH method with $L_{1/2}$ penalty (denote as $L_{1/2}$-RCPH) selects the least number of genes, while the Ridge-RCPH method selects the largest one because it causes no zero coefficients [22]. Specifically, the Ridge-RCPH method achieves the highest C-index, the Enet-RCPH method is the run-up, and the MCP-RCPH method obtains the lowest one. What’s more, the *P*-value of the $L_0$-RCPH method is the smallest and that of the MCP-RCPH method is the highest. Based on the above two indexes, Enet-RCPH achieves the superior performance with the C-index approximately equal to 0.800 and *P*-value lower than 0.050, followed by $L_0$-, SCAD- and Lasso-RCPH respectively. And they reach the better performances when compared with the other three methods.

**Fig. 2:**
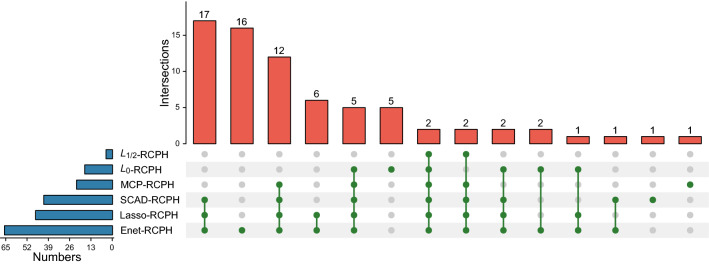
The overlap of features among the five different RCPH methods (except for Ridge-RCPH method), where the top bar shows that the interactions among different methods described by bottom dotted lines

Figure 2 illustrates the overlapping summary statistics of these feature genes selected by the six RCPH models. We find the Enet-RCPH method selects out the most feature genes, and it has a large number of intersections with those of SCAD- and Lasso-RCPH. In order to identify robust biomarkers, we select four optimal feature subsets (i.e., the four gene sets identified by Lasso-, Enet-, $L_0$- and SCAD-RCPH) based on the principle of large C-index and small *P*-value (C-index $\ge 0.700$ & *P*-value $\le 0.050$). We take the union 72 genes as the detected biomarker genes.

### Literature validation

We validate these detected prognosis biomarkers of BRCA against the literature report of signature genes. Interestingly, numerous researches have investigated the relationship between these genes and BRCA. Among the 72 genes we identified, 51 genes have been confirmed in the literature that they are indeed related to the occurrence and prognosis of BRCA. The detailed list can be found in Additional file 2: Table S2.

Particularly, the rest 21 biomarker genes that have not been reported in the literature so far and they are valuable to be confirmed by further experiments. Though their clinical significance is not clear, they can be potentially novel signatures for human BRCA. They are novel prognostic biomarkers and beneficial to the target development for precision therapy.

### Breast cancer-specific gene regulatory network (BRCA-specific GRN)

**Fig. 3:**
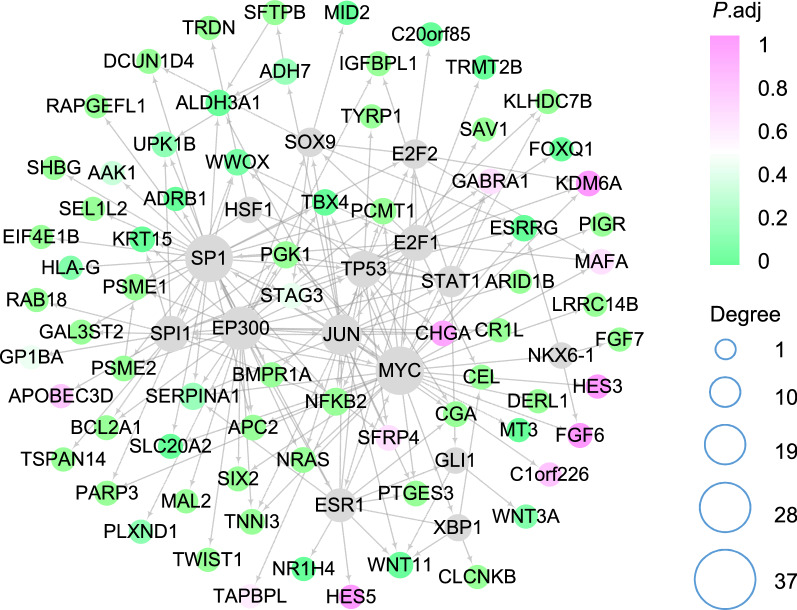
The network structure of 72 biomarkers, where the color shows the significance of gene expression difference between normal and disease, and the node size refers to its degree

In the 1142 candidates, we firstly build up an underlying specific GRN of BRCA according to the prior background network documented in RegNetwork [29] and high gene co-expression by Pearson’s correlation coefficient and mutual information [44]. The specific network is with 6402 edges as shown in Additional file 3: Table S3. Intuitively, Fig. 3 visualizes the extracted network structure underlying 72 identified biomarker genes.

As shown, we can see that most of the identified biomarkers (51 out of 72) are significantly differentially expressed ($P<0.05$) , which confirms that DEGs are likely to be the signatures that play important roles in the occurrence and development of BRCA. Note that the 15 genes represented by gray nodes are not included in the 72 biomarkers. However, they are of great significance because all of them have regulatory interactions with 72 biomarkers. Especially, *TP53*, *E2F1*, *SP1*, *MYC* and *JUN* have been revealed to be genes related to BRCA [15]. Although the identified biomarkers do not contain these 15 genes, many biomarker genes are regulated by them. The network structure indicates the biomarkers selected by RCPH methods are related to BRCA.

### Functional implications

To explore the potential pathological implications of these biomarker genes for BRCA, we also perform GO functional enrichment analysis. Firstly, we perform the GO enrichment analysis on the 72 genes and obtain 23 significantly enriched GO biological processes. We find the knowledge-based BRCA dysfunctions are enriched in these identified biomarkers. This verifies the functional implications of biomarker genes. In turn, they prove the effectiveness of our proposed RCPH models (5) in biomarker discovery.

Second, we calculation the SS-measure value [42] between these enriched GO terms and some unique GO terms in BRCA obtained by hallmarks of cancer [41]. The detailed results are shown in Additional file 4: Table S4. Specifically, we randomly pick out 23 terms from all cancer-related GO terms and calculate the SS-measure between them and the former enriched GO terms. Without loss of generality, we repeat this process dozens of times and take the average value as the final similarity value, which greatly improves the generalization performance of this kind of measure.

**Fig. 4:**
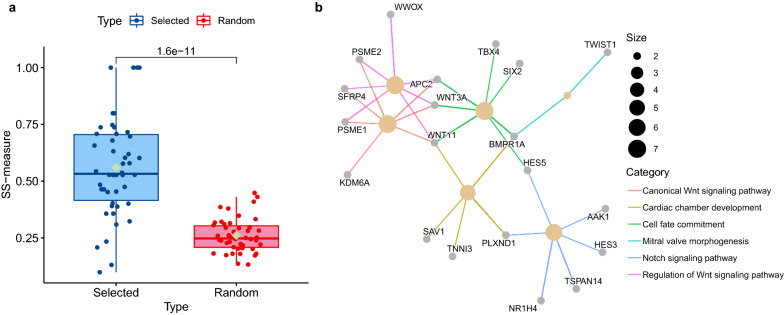
SS-measure boxplots and significantly enriched functions in the 72 biomarker genes. **a** The SS-measure between our enriched GO terms and randomly selected GO terms by comparing with the unique GO terms in hallmarks of BRCA. **b** The gene network that closely related to the top 6 significant GO terms with SS-measure $\ge 0.75$ of our enriched functions

Consequently, a significantly higher value of SS-measure is observed in enriched GO terms than in random GO terms (*P* = 1.6e−11, Wilcoxon test) as shown in Fig. 4a. The results indicate the functions enriched in the 72 identified biomarkers are indeed related to BRCA. Figure 4b shows the top 6 enriched GO biological process (BP) terms with higher SS-measure ($\ge 0.75$) and their related genes in the form of a network. These enriched functions have been involved in BRCA progression, which provides evidence for further implications of the molecular mechanisms of our discovered biomarkers [45].

**Fig. 5:**
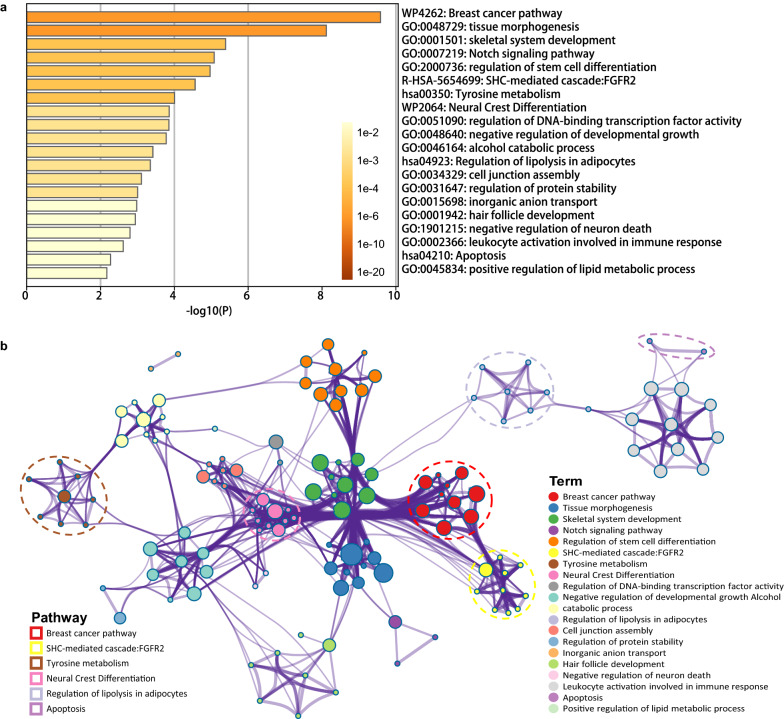
Pathway enrichment analysis of 72 biomarker genes, where the *P*-value is calculated by accumulative hypergeometric test. **a** The heatmap of top 20 statistically enriched terms. **b** The functional enrichment map of pathways, where the subsets of representative terms are selected from the cluster and converted into a network layout. More specifically, each circle node represents a term, where its size is proportional to the number of input genes that fall into that term, and its color represents its cluster identity (i.e., nodes of the same color belong to the same cluster)

To further study the biological functions underlying the 72 biomarkers, we also performed pathway enrichment analysis. Figure 5a gives a heatmap of the top 20 enriched clusters, which is colored according to *P*-value. It can be seen that the most significant pathway is ‘WP4262: Breast cancer pathway ($P\approx$ 1.0e-10)’. Figure 5b shows the functional enrichment map of these pathways, where each node represents an enriched term, the node size represents the number of genes in the pathway. It shows that the pathways enriched are almost BRCA-related pathways, e.g., tissue morphogenesis and skeletal system development [46, 47].

### PRS system construction and internal validation

To build a risk scoring system based on the identified signatures of BRCA, the 72 feature genes of 1080 patients with clinical information from TCGA are reserved for further analysis. As shown in Additional file 5: Table S5, univariate and multivariate Cox regression analysis of each for OS is conducted to select several key prognostic genes so as to construct the PRS model.

Firstly, univariate Cox regression survival analysis is performed to discover candidate signatures, and the 13 genes with $P<0.05$ are considered as significantly correlated (4 genes positively correlated and 9 genes inversely correlated) with survival. Subsequently, multivariate Cox regression is conducted to select independent prognostic genes with $P<0.05$ associated with survival. Eventually, three genes (*ADRB1*, *SAV1* and *TSPAN14*) are proved to be (inversely) highly correlated with OS. Thus, we propose the PRS index, which can be regarded as a three-gene signature model

12 $$\begin{aligned} \text {PRS} = -0.068 * x_{ADRB1} -0.090 * x_{SAV1} -0.105 * x_{TSPAN14}, \end{aligned}$$

where *x* represents the expression value of its corresponding gene. The optimal cut-off value is automatically generated by X-Tile [48, 49]. Subsequently, all patients can be categorized into a high-risk group and a low-risk group via the optimal cut-off value of PRS.

**Fig. 6:**
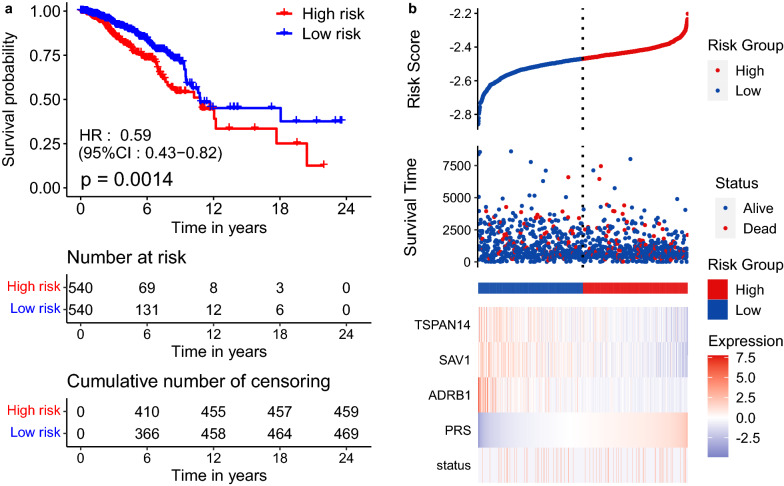
The association between PRS index and overall survival status in BRCA patients on the discovery dataset. **a** The KM-curves of PRS. **b** The distributions of PRS and survival time for each sample

To justify the PRS system associated with BRCA, we validate our findings in the discovery dataset, i.e., internal validation. The expression profiles of the three genes in Eq.(12) are extracted from TCGA. After submitting them into Eq.(12), the PRS index is obtained. Using the median of PRS to divide the BRCA patients into a high-risk group and low-risk group, the KM-curve can be derived as shown in Fig. 6a. It is clear that there are significant differences between the two patient groups on the survival probability ($P = 0.0014$, Log-rank test).

Moreover, we calculate the PRS for each sample individually on the entire dataset to draw the PRS distribution, as shown in Fig. 6b. Compared with low PRS, the samples with high PRS have poorer prognoses. It can be seen that in different samples, the different expression of the three biomarker genes increases the PRS index. Therefore, the high expression value of these genes can be identified as the potential risk factor for BRCA prognosis. The built-up PRS system obtains a better survival analysis in the internal validation dataset. It proves the effectiveness of the PRS index in the prognosis prediction of BRCA.

### Nomogram construction and internal validation

**Fig. 7:**
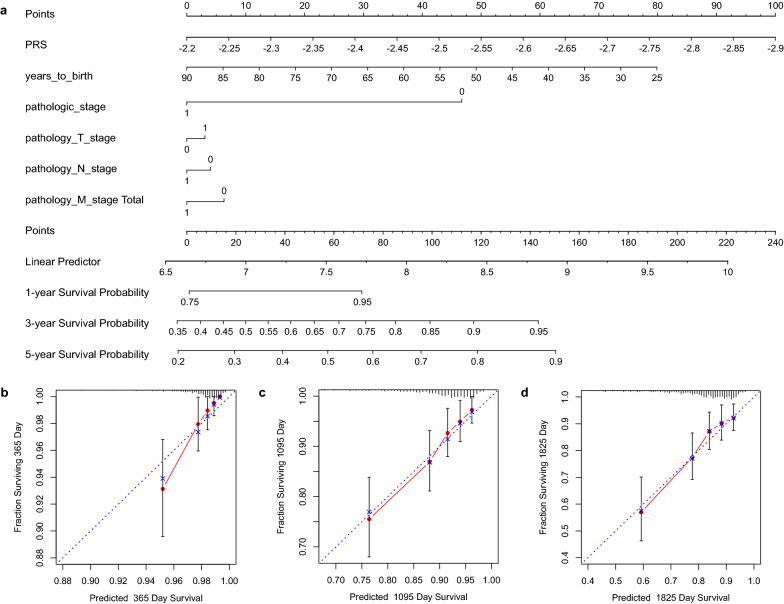
The nomogram for predicting the proportion and the calibration plots in terms of the agreement between predicted of BRCA patients. **a** The nomogram for predicting proportion of BRCA patients with 1-year, 3-year and 5-year, respectively. **b**–**d** The calibration plots for predicting 1-year, 3-year and 5-year

We first construct the grouping variables (where I and II are 0, III, IV and V are 1) and then plot nomogram on the discovery dataset as shown in Fig. 7a, where the length of the line segment reflects the contribution of this factor to the final event, and lower total points indicate a worse outcome. Clearly, PRS has the greatest impact on the outcome, and age has the second greatest impact, which is consistent with the fact found in literature [50]. Often, the risk for BRCA increases with age, and most BRCA patients are diagnosed after age 50. The length of the line segment induced by the PRS index is a little longer than the age index, which indicates that the estimation of the survival probability by PRS is quite important. The third most affected factor is the pathologic stage, and the contrast is more obvious than the rest three clinical factors.

Figure 7b–d shows the calibration figures in terms of the agreement between the prediction of BRCA patients for 1-, 3- and 5-years. For the calibration curve, the *x*-axis represents the nomogram survival prediction probability, the *y*-axis represents the actual survival probability, and the diagonal line of the blue dot represents the best prediction. The higher the coincidence degree between the red fit line and the blue diagonal line, the better performance of the nomogram exhibits [8]. Compared with the ideal model, the nomogram has a better prediction effect, especially for the 3-year and 5-year OS estimations. In general, Fig. 7 highlights the PRS indicator has important guiding significance for the prognosis of BRCA patients.

### Prognosis and prediction of PRS

**Fig. 8:**
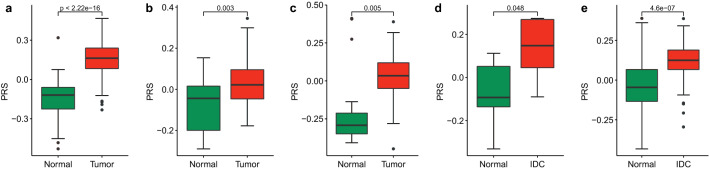
The comparison of PRS index in normal and tumor tissues in the internal TCGA dataset and four external GEO datasets. **a** TCGA (*n* = 224). **b** GSE7904 (*n* = 62). **c** GSE42568 (*n* = 121). **d** GSE5764 (*n* = 15). **e** GSE10780 (*n* = 185)

To further explore the performance of PRS in prognosis and prediction, the values of PRS in normal and tumor tissues of BRCA are evaluated in the internal TCGA dataset and four external GEO datasets. As shown in Fig. 8, the PRS in tumor tissues are significantly higher than those in normal tissues. It shows that the PRS index we proposed has a very important indicator for the prognosis and prediction of BRCA.

**Fig. 9:**
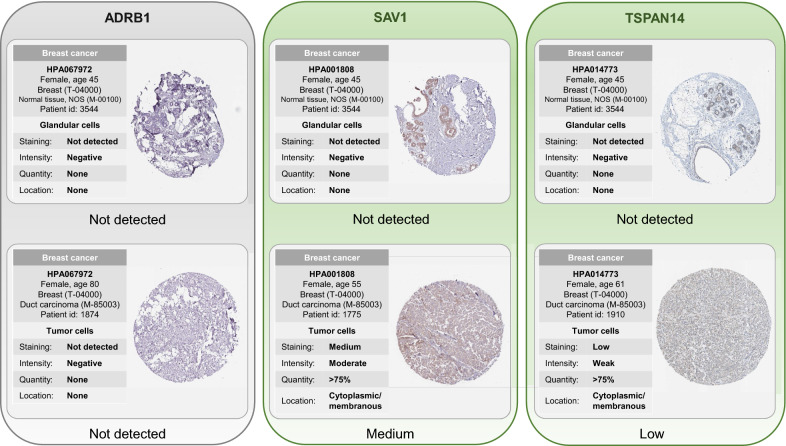
Protein expression profiles of the three genes (ADRB1, SAV1 and TSPAN14) in PRS between normal and tumor tissues. All subfigures are extracted from the Human Protein Atlas database

Consistently, we further explore the protein expression profiles of the three PRS genes in Eq.(12) from the Human Protein Atlas (https://www.proteinatlas.org/) database. For the results of certain protein immunohistochemistry, they are divided into four categories: Not detected, Low expression, Medium expression, and High expression. The images in Fig. 9 show the detailed immunohistochemical staining in normal breast tissue and tumor tissue, respectively. As shown, *SAV1* and *TSPAN14* are more strongly stained in the tumor specimens, and there are kinds of literature showing that their expression profiles are confirmed to be related to BRCA [51, 52]. There is no significant difference in the expression values of *ADRB1* between normal and tumor specimens of BRCA. However, we found that it has just been defined as a potential biomarker for BRCA by the co-analysis of tumor mutational burden and immune infiltration [53].

**Fig. 10:**
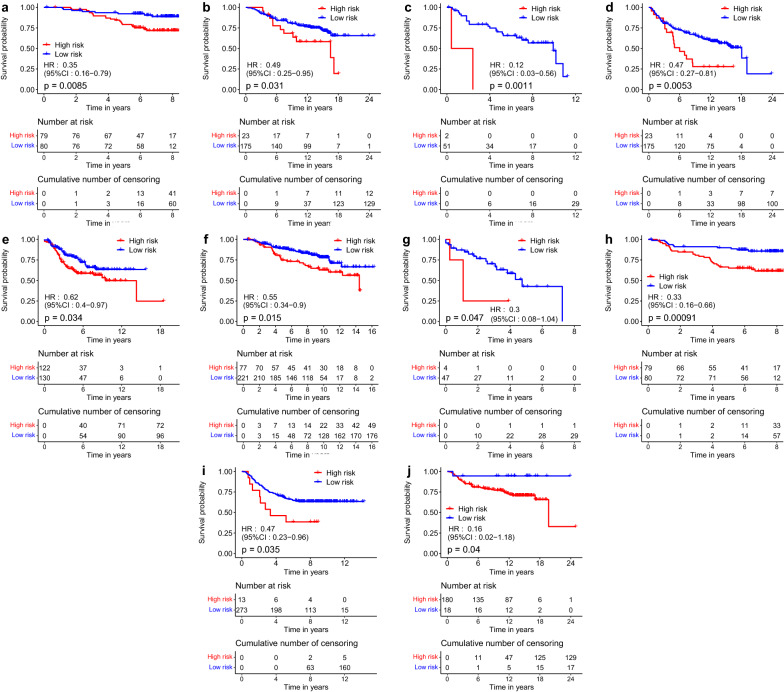
The KM-curves of high-risk and low-risk BRCA patients in some external datasets from the GEO database. **a** GSE1456 (OS). **b** GSE7390 (OS). **c** GSE35629 (OS). **d** GSE7390 (DFS). **e** GSE21653 (DFS). **f** GSE17705 (RFS). **g** GSE35629 (RFS). **h** GSE1456 (DMFS). **i** GSE1456 (DMFS). **j** GSE7390 (TDM)

### External validation

In order to show the effectiveness of the proposed PRS system, we verify it on some external datasets from GEO. Similar to the process of internal validation, we firstly extract the three genes in Eq.(12) from the 10 independent datasets listed in Table 1. Then we apply the expression profiles of three genes from each sample to compute the PRS index. Next, we use the optimal cut-off value of PRS to categorize the patients into a high-risk group and low-risk group and apply the Log-rank test to compute *P*-value on each independent external validation dataset respectively. In total, 1848 patient samples are obtained for external validation. The detailed results (KM-curve, hazard ratio (HR), $95\%$ confidence interval ($95\%$ CI) and *P*-value) are shown in Fig. 10. From the figures, we can easily find that the survival curve based on the PRS index is significantly different on each independent dataset.

Note that Fig. 10 contains more than one kind of information about survival. This is because BRCA patients often have more than one important tumor clinical trial endpoint from diagnosis to remission or death. For example, OS in Fig. 10a–c refers to the time from the beginning to death from any cause, DFS in Fig. 10d–e refers to the time from random start to disease recurrence or patient death due to disease progression. A detailed explanation of other survival times (e.g., RFS in Fig. 10f–g, DMFS in Fig. 10h–i and TDM in Fig. 10j) can be found in the original dataset, respectively. Thus, from the perspective of survival time, we actually perform survival analysis on 1848 BRCA patients from 10 independent external validation datasets. So far, they are one of the largest BRCA cohorts based on gene expression data. The results indicate the PRS system we established is significantly effective for the prognosis of BRCA.

## Discussion

Prognostic biomarkers of BRCA will not only affect the incidence but also influence its recurrence and survival to a large extent [54]. In this paper, we discovered biomarkers (risk genes) related to the prognosis of BRCA by using RCPH models from gene expression data. We also verified these identified biomarkers in a total of 3535 BRCA samples (including the discovery dataset and external verification datasets). We found these prognostic biomarker genes form a BRCA-specific GRN with dysfunctional indicators. Based on identified biomarkers, we also developed a scoring system (PRS model) that assesses the prognosis and survival status of BRCA patients. This is of great significance for estimating potential risks, enriching prognostic biomarkers, and developing a detection system of BRCA.

Different from the available methods of discovering biomarkers, our work is proposed to find prognostic biomarkers based on the combination of prior knowledge and large-scale gene expression data. Moreover, we established an effective PRS model based on the interpretable ensemble feature selection strategy. First, unlike the method of Tao et al. [55], we not only use differential gene expression analysis to screen out feature genes from all genes (i.e., dimension reduction). Considering the important disease-causing genes are not always differentially expressed, we also integrated prior information of multiple cancer pathways, which laid the foundation for more accurate biomarker discovery. Second, in contrast with the weighted gene co-expression network analysis (WGCNA) method of Yan et al. [8], we did not use GRN to select important hub genes with the largest module size as potential prognostic genes. Based on the linkages between genes and guided by regularization models, we employed ensemble feature selection methods with RCPH methods to identify possible BRCA prognostic genes in the form of GRN. It not only selects the risk genes explainably, but also describes the integrative dysfunctional pathway underlying these selected genes. Finally, different from the work of Chen et al. [45] and Zhou et al. [56], instead of applying Cox regression to each DEG for prognostic analysis, we developed a PRS model based on a set of feature genes. Based on the fact that a single gene often leads to an unstable prognosis, the discovered risk genes are insufficient in revealing complex molecular mechanisms [57]. In contrast, the feature selection is performed via an interpretable dimensionality reduction process of removing redundant factors from thousands of gene candidates. The selected genes are meaningful to serve the subsequent establishment of the PRS index. We applied RCPH models on this basis that greatly reduced the computational cost and improved the accuracy of prediction.

Our results suggest that in the univariate and multivariate Cox regression analysis, *ADRB1*, *SAV1* and *TSPAN14* were significantly related to the prognosis of BRCA patients ($P<$0.05). While *ADRB1* is different from what we have observed in the protein expression profiles. Its expression value is not significantly different between normal breast specimens and tumor specimens. Interestingly, we found that the work of Wang et al. [53] has just identified it as a potential biomarker for BRCA, which reflects the potential value of our findings of biomarker genes for BRCA. In summary, we have identified 72 prognostic biomarkers of BRCA by RCPH models and selected three of them (*ADRB1*, *SAV1* and *TSPAN14*) to develop a PRS system. Survival analysis confirmed that the PRS model exhibits significant predictive ability and prognostic value for BRCA patients with different stages.

For how the DEGs are related to chemoresistance. We know that BRCA, especially the aggressive subtype triple-negative breast cancer, frequently develops resistance to chemoresistance. Particularly, as shown in Additional file 6: Figure S1, there are nine genes contained in the intersection of the four feature subsets identified by Lasso-RCPH, Enet-RCPH, L0-RCPH and SCAD-RCPH respectively. Here, we only focused on six DEGs among the nine overlapped genes and analyzed the relationship between those six DEGs and BRCA chemoresistance. The results are shown in Table 5. As shown, four genes have been confirmed in the literature to be significantly related to BRCA chemoresistance.

**Table 5:** The relationship between six DEGs and chemoresistance

Gene symbol	Description	References
CEL	Carboxyl Ester Lipase	–
PGK1	Phosphoglycerate Kinase 1	[80–83]
PTGES3	Prostaglandin E Synthase 3	–
RAPGEFL1	Rap Guanine Nucleotide Exchange Factor Like 1	[84]
SERPINA1	Serpin Family A Member 1	[85, 86]
WWOX	SWW Domain Containing Oxidoreductase	[87–89]

There are still some limitations in our current study. On the one hand, our proposed method only worked at the gene expression level. Multiple omics data, e.g., DNA methylation [52], copy number variation [58], protein complexes [57] and metabolite information [59] will provide integrative and comprehensive information for biomarker discovery. The integration of multi-omics data is an important approach for discovering more accurate BRCA biomarkers. On the other hand, for the selected 72 biomarker genes, we only employed literature validations and computational methods for preliminary verification. They are expected to be justified via *in vitro* experiments and clinical trials for BRCA patients, which is critically important for translational research and clinical validation/application. These are the research directions in the future. Currently, this paper is majorly focusing on the RCPH models of identifying potential biomarker genes. Obviously, the proposed framework is rather general to be easily extended to discover prognostic biomarkers for other complex diseases.

Discovery, verification and qualification have become standard procedures commonly used to obtain high-quality biomarkers. Our research is dedicated to the discovery and preliminary verification stages, the subsequent clinical *in vitro* verification and further qualification to re-identify the biomarkers are very important to determine the biomarkers that can finally be transformed into clinics. In the new era, one of the core tasks of translational medicine is to establish a low-cost, high-throughput, and highly reproducible technical system. Currently, the Stable Isotope Standards and Capture by Anti-Peptide Antibodies (SISCAPA) technology developed by Anderson et al. [60] can effectively bridge the gap between the discovery and clinical application of biomarkers. It has important reference significance for the translational medicine of biomarker research. In the future, we plan to carry out medical practice based on the needs of patients, use SISCAPA technology to bring the identified biomarkers into clinics. We will strive to provide more accurate early-warning, diagnosis and treatment of BRCA by translating the screened biomarkers into clinics.

## Conclusion

In this paper, we proposed a method of detecting prognostic biomarkers of BRCA from gene expression data by RCPH models. Compared with the focus only on individual genes, our method makes full use of data-driven by considering the expression profiles, known potential candidates, and the networking interconnection between genes. The RCPH models guarantee the ensemble feature selection of potential biomarkers by screening candidate genes, and the built-up PRS system seeks and verify genuine prognosis biomarkers of BRCA.

For achieving fair comparisons, we compared these RCPH models with seven penalties (i.e., Ridge, Lasso, Elastic net, $L_0$, $L_{1/2}$, SCAD, and MCP) based on their performance metrics of C-index and *P*-value on the biomarker discovery dataset. By integrating the feature genes selected in the models with the top performances, we identified 72 robust prognostic biomarkers of BRCA. We validated the findings from various aspects. Firstly, most biomarkers genes have been confirmed in the relationship with BRCA in the literature validation. Then, we established a specific GRN of BRCA and visualized the network module of these 72 biomarkers. They are consistent with known disease genes or neighbor genes. At last, the enriched functions underlying these selected biomarkers indicated their important implications in BRCA.

More importantly, we constructed a PRS system by employing univariate and multivariate Cox regression models based on these biomarker genes. Then we validated the effectiveness and efficiency of the PRS system in the 1080 BRCA samples from internal validation data and in the 1848 BRCA samples from external independent validation data. We also explored the difference between the PRS value and the protein expression profiles of the PRS genes involved in tumor samples and controls. The results indicated PRS is expected to be an important indicator for the prognosis of BRCA patients.

## Supplementary information

**Additional file 1: Table S1.** The DEGs and the breast cancer-related genes selected from KEGG, GO terms, MammaPrint, OSbrca and scPrognosis.

**Additional file 2: Table S2.** There 51 genes have been confirmed in literature that they are indeed related to the occurrence and prognosis of breast cancer, and the remaining 21 genes have not been confirmed.

**Additional file 3: Table S3.** The breast cancer-specific gene regulatory network with 1142 genes and 6402 edges.

**Additional file 4: Table S4.** The 23 significantly enriched GO terms and some GO terms unique in breast cancer obtained from cancer hallmarks.

**Additional file 5: Table S5.** The results of univariate and multivariate Cox regression analysis on 72 identified biomarkers.

**Additional file 6: Figure S6.** The overlap genes in the four feature subsets identified by Lasso-RCPH, Enet-RCPH, *L*_0_-RCPH and SCAD-RCPH.

## Data Availability

Not applicable (The current study was performed based on published literature and no datasets were generated. All data analyzed and used during this study can be found in TCGA in database and GEO database).

## Abbreviations

RCPH: Regularized Cox proportional hazards
DEGs: Differentially expressed genes
GRN: Gene regulatory network
PRS: Prognostic risk score
OSbrca: Online consensus Survival analysis web server for Breast Cancer
GO: Gene ontology
CPH: Cox proportional hazard
Enet: Elastic net
TCGA: The Cancer Genome Atlas
GEO: Gene Expression Omnibus
*P*.adj: Adjusted *P*-value
IQR: Interquartile range (1,3)
OS: Overall survival
DMFS: Distant metastasis-free survival
TDM: Time to distant metastasis
DFS: Disease-free survival
RFS: Relapse free survival
GOA: Gene ontology annotation
IDC: Invasive ductal carcinomas
ILC: Invasive lobular carcinomas
CV: Cross-validation
CV-LPL: Cross-validation log partial likelihood
C-index: Concordance index
KM: Kaplan-Meier
SS-measure: Semantic similarity measure
HR: Hazard ratio
$95\%$ CI: $95\%$ confidence interval
WGCNA: Weighted gene co-expression network analysis
SISCAPA: Stable Isotope Standards and Capture by Anti-Peptide Antibodies
