# Supplementary material for: Detecting prognostic biomarkers of breast cancer by regularized Cox proportional hazards models
Source: J Transl Med. 2021 Dec 20;19:514. doi: 10.1186/s12967-021-03180-y (PMC8686664; doi:10.1186/s12967-021-03180-y)
Supplement: Supplementary file 2 — Additional file 2: Table S2. There 51 genes have been confirmed in literature that they are indeed related to the occurrence and prognosis of breast cancer, and the remaining 21 genes have not been confirmed. [file 12967_2021_3180_MOESM2_ESM.pdf]

S1 Table. **The genes that have been reported in the works of literature.** The 51 genes have been confirmed in the works of literature to be indeed related to the occurrence and prognosis of breast cancer.

Biomarker	Description	Reported literatures	KEGG pathway	GWAS Catalog	Gene ORGA-Nizer
ADRB1	ADRB1 is identified as a potential biomarker for breast cancer by the co-analysis of tumor mutational burden and immune infiltration.	[1]			
ALDH3A1	Cellular levels of ALDH3A1 as predictors of therapeutic responses to cyclophosphamide-based chemotherapy of breast cancer.	[2]	✓		
APOBEC3D	APOBEC3D is differentially expressed in the lymph nodes of patients with metastatic breast cancer.	[3]			
ARID1B	ARID1B is lost in breast cancer.	[4]			✓
BCL2A1	KCNN4 induces multiple chemoresistance in breast cancer by regulating BCL2A1.	[5, 6, 7]			
BMPR1A	Knockdown of BMPR1A of breast cancer cells suppresses their production of RANKL via p38 pathway and inhibits cancer-induced osteoclastogenesis.	[8, 9, 10]		✓	
C1orf226	C1orf226 is a downregulated gene in the Triple-Negative Breast Cancer (TNBC) tissues compared to normal breast tissues.	[11, 12]			
CGA	The CGA gene as new predictor of the response to endocrine therapy in ERα-positive postmenopausal breast cancer patients.	[13]	✓		
CHGA	Diseases associated with CHGA include acinar cell carcinoma and tubular adenocarcinoma. Among its related pathways are defensins and innate immune system.	[14, 15]			
DCUN1D4	An important paralog of DCUN1D4 is DCUN1D5, DCUN1D5 is significantly hypermethylated in the parous breast.	[16]			
DERL1	DERL1 mRNA overexpression correlates with positive lymph node metastasis in breast cancer.	[17]			
ESRRG	miR-378 targets endogenous ESRRG and GABPA and reduces their expression in human breast cancer cells.	[18]	✓		
FGF6	FGF6 amplificates in human breast-tumors and increases cell death in breast cancer cell lines.	[19]	✓	✓	✓
FGF7	The fibroblast growth factor binding protein is a novel interaction partner of FGF7 and regulates FGF activity: implications for epithelial repair.	[20, 21, 22]	✓		
FOXQ1	FOXQ1 is differentially expressed across breast cancer subtypes with low expression associated with poor overall survival.	[23, 24, 25, 26]			
GAL3ST2	GAL3ST2 is present in NMMuMG and two human breast cancer cell lines, and it is more strongly expressed in more metastatic tumors.	[27]			
GP1BA	the gene expression of GP1BA is shared by all transcriptomic subgroups of breast cancer, suggesting that there is a gene expression program that is common to all breast metastases.	[28]			

Biomarker	Description	Reported literatures	KEGG pathway	GWAS Catalog	Gene ORGA-Nizer
HES3	The anti-tumor efficacy of an enzyme involved in Notch receptor activation can be predicted by the level of expression of HES3 in breast cancer xenograft models.	[29, 30]			
HES5	HES5 is a disease-specific gene in breast cancer.	[31]	✓		
HLA-G	HLA-G expression in classical HLA class I-negative tumors is of prognostic value for clinical outcome of early breast cancer patients.	[32, 33, 34, 35]			
IGFBPL1	Insulin-like growth factor binding protein (IGFBP)-related IGFBPL1 has an independent function in suppressing breast cancer phenotype.	[36, 37, 38]			
KDM6A	Chemotherapy-induced S100A10 recruits KDM6A to facilitate OCT4-mediated breast cancer stemness.	[39, 40]			✓
KLHDC7B	A Kelch domain-containing KLHDC7B acts oppositely on breast cancer cell proliferation via the interferon signaling pathway.	[41]			
KRT15	Low KRT15 expression is associated with poor prognosis in patients with breast invasive carcinoma	[42]			
MAL2	MAL2 drives immune evasion in breast cancer by suppressing tumor antigen presentation.	[43, 44]			
MID2	MORC4 promotes chemoresistance of luminal A/B breast cancer via STAT3-mediated MID2 upregulation.	[45]			
MT3	Increase in fluidity in the membrane of MT3 breast cancer cells correlates with enhanced cell adhesion in vitro.	[46]			
NFKB2	Diseases associated with NFKB2 include immunodeficiency, common variable, 10 and common variable immunodeficiency.	[47, 48]	✓		
NR1H4	NR1H4 is a member of the nuclear receptor superfamily of ligand-dependent transcription factors, it has been recently detected in breast cancer cell lines and tissue specimens.	[49, 50, 51, 52]			
NRAS	MicroRNA-22 suppresses breast cancer cell growth and increases paclitaxel sensitivity by targeting N-RAS.	[53, 54]			✓
PARP3	PARP3 inhibitors ME0328 and olaparib potentiate vinorelbine sensitization in breast cancer cell lines.	[55]	✓		
PCMT1	Breast cancer patients with higher PCMT1 expression have significantly lower survival rates than those with lower PCMT1 expression.	[56]			
PGK1	PGK1 is a potential survival biomarker and invasion promoter by regulating the HIF-1α-Mediated epithelial-mesenchymal transition process in breast cancer.	[57, 58, 59]			
PIGR	The immune function-related gene PIGR is detected as downregulated in breast cancer.	[60]	✓		
PLXND1	PLXND1 is ubiquitously expressed on tumor vessels and tumor cells in solid malignancies.	[61, 62]			✓
PTGES3	Genes such as JAK2, TBP, PTGES3, and RYBP may be promising prognostic biomarkers for breast cancer patients.	[63]			

Biomarker	Description	Reported literatures	KEGG pathway	GWAS Catalog	Gene ORGA-Nizer
RAB18	Several microarray studies have shown dysregulation of RAB18 in breast cancer.	[64, 65]			
RAPGEFL1	The ERGs RAPGEFL1 is identified ceRNAs of NEAT1, the role of NEAT1 in breast cancer has been widely investigated.	[66]			
SAV1	SAV1 expression is found to be downregulated in a variety of cancers, including breast cancer.	[67]			
SERPINA1	SERPINA1 is a direct estrogen receptor target gene and a predictor of survival in breast cancer patients.	[68]			
SFRP4	Inhibition of breast cancer stem-like cells by a triterpenoid, ursolic acid, via activation of Wnt antagonist and SFRP4.	[69]	✓	✓	✓
SHBG	Decrease of circulating level of SHBG in postmenopausal obese women as a risk factor in breast cancer.	[70]			
SIX2	Homeoprotein SIX2 promotes breast cancer metastasis via transcriptional and epigenetic control of E-cadherin expression.	[71, 72]			
STAG3	Investigating STAG3 expression as a novel human breast cancer Biomarker.	[73, 74]			
TAPBPL	TAPBPL protein was detected in normal breast tissue at low levels, as compared to isotype antibody staining .	[75]	✓		
TSPAN14	The average methylation difference 10% between breast cancer patients and controls mapped to gene TSPAN14, which is previously described as cancer genes.	[76]			
TWIST1	TWIST1-mediated adriamycin-induced epithelial-mesenchymal transition relates to multidrug resistance and invasive potential in breast cancer cells.	[77, 78, 79, 80]	✓		✓
TYRP1	Expression of tyrosinase-related protein 1 (TYRP1) correlates with the absence of metastasis in an isogenic human breast cancer model.	[81]	✓		
WNT11	WNT11 is a novel ligand for ROR2 in human breast cancer.	[82, 83]	✓		
WNT3A	MiR-6838-5p suppresses cell metastasis and the EMT process in triple-negative breast cancer by targeting WNT3A to inhibit the Wnt pathway.	[84, 85]	✓		
WWOX	WWOX is a novel WW domain-containing protein mapping to human chromosome 16q23.3-24.1, a region frequently affected in breast cancer.	[86, 87, 88]		✓	✓

S1 Table Continued. **The genes that haven't been reported in the works of literature.** The 21 genes haven't been confirmed in the works of literature to be indeed related to the occurrence and prognosis of breast cancer.

Biomarker	Description	Reported literatures	KEGG pathway	Gene ORGA-Nizer
AAK1				
ADH7				
APC2				
C20orf85				
CEL				
CLCNKB				
CR1L				
EIF4E1B				
GABRA1				
LRRC14B				
MAFA				
PSME1	Diseases associated with PSME1 include Immunodeficiency 12.			
PSME2	Diseases associated with PSME2 include Immunodeficiency 12.			
SEL1L2				
SFTPB				
SLC20A2				
TBX4				
TNNI3				
TRDN				
TRMT2B				
UPK1B				

References

[1] J. Wang, X. Zhang, J. Li, X. Ma, F. Feng, L. Liu, J. Wu, C. Sun, Adrb1 was identified as a potential biomarker for breast cancer by the co-analysis of tumor mutational burden and immune infiltration, Aging (Albany NY) 13 (2021) 351.

[2] N. E. Sládek, R. Kollander, L. Sreerama, D. T. Kiang, Cellular levels of aldehyde dehydrogenases (aldh1a1 and aldh3a1) as predictors of therapeutic responses to cyclophosphamide-based chemotherapy of breast cancer: a retrospective study, Cancer chemotherapy and pharmacology 49 (2002) 309–321.

[3] S. Mamoor, Apobec3d is differentially expressed in the lymph nodes of patients with metastatic breast cancer. (2021).

[4] T. Aso, H. Uozaki, S. Morita, A. Kumagai, M. Watanabe, Loss of arid1a, arid1b, and arid2 expression during progression of gastric cancer, Anticancer research 35 (2015) 6819–6827.

[5] P. Lin, J. Li, F. Ye, W. Fu, X. Hu, Z. Shao, C. Song, Kcnn4 induces multiple chemoresistance in breast cancer by regulating bcl2a1, American journal of cancer research 10 (2020) 3302.

[6] M. Hiraki, T. Maeda, N. Mehrotra, C. Jin, M. Alam, A. Bouillez, T. Hata, A. Tagde, A. Keating, S. Kharbanda, et al., Targeting muc1-c suppresses bcl2a1 in triple-negative breast cancer, Signal transduction and targeted therapy 3 (2018) 1–8.

[7] B. Yu, W. You, G. Chen, Y. Yu, Q. Yang, Mir-140-5p inhibits cell proliferation and metastasis by regulating muc1 via bcl2a1/mapk pathway in triple negative breast cancer, Cell cycle 18 (2019) 2641–2650.

[8] E.-L. Alarmo, T. Kuukasjärvi, R. Karhu, A. Kallioniemi, A comprehensive expression survey of bone morphogenetic proteins in breast cancer highlights the importance of bmp4 and bmp7, Breast cancer research and treatment 103 (2007) 239–246.

[9] Y. Liu, R.-X. Zhang, W. Yuan, H.-Q. Chen, D.-D. Tian, H. Li, X. Jiang, Z.-L. Deng, Y. Wang, Knockdown of bone morphogenetic proteins type 1a receptor (bmpr1a) in breast cancer cells protects bone from breast cancer-induced osteolysis by suppressing rankl expression, Cellular physiology and biochemistry 45 (2018) 1759–1771.

[10] M. W. Pickup, L. D. Hover, Y. Guo, A. E. Gorska, A. Chytil, S. V. Novitskiy, H. L. Moses, P. Owens, Deletion of the bmp receptor bmpr1a impairs mammary tumor formation and metastasis, Oncotarget 6 (2015) 22890.

[11] T. Chuan, T. Li, C. Yi, Identification of cxcr4 and cxcl10 as potential predictive biomarkers in triple negative breast cancer (tnbc), Medical science monitor: international medical journal of experimental and clinical research 26 (2020) e918281–1.

[12] R. Cheng, L. Qi, X. Kong, Z. Wang, Y. Fang, J. Wang, Identification of the significant genes regulated by estrogen receptor in estrogen receptor-positive breast cancer and their expression pattern changes when tamoxifen or fulvestrant resistance occurs, Frontiers in genetics 11 (2020).

[13] I. Bièche, B. Parfait, C. Noguès, C. Andrieu, D. Vidaud, F. Spyratos, R. Lidereau, M. Vidaud, The cga gene as new predictor of the response to endocrine therapy in erα-positive postmenopausal breast cancer patients, Oncogene 20 (2001) 6955–6959.

[14] L. Annaratone, E. Medico, N. Rangel, I. Castellano, C. Marchiò, A. Sapino, G. Bussolati, Search for neuro-endocrine markers (chromogranin a, synaptophysin and vgf) in breast cancers. an integrated approach using immunohistochemistry and gene expression profiling, Endocrine pathology 25 (2014) 219–228.

[15] S. Seltzer, M. Corrigan, S. O'Reilly, The clinicomolecular landscape of de novo versus relapsed stage iv metastatic breast cancer, Experimental and molecular pathology 114 (2020) 104404.

[16] S. Zhou, S. Guan, X. Lv, R. Ma, Y. Zhang, Y. Yan, J. Wang, X. Sun, M. Gao, D. Di, et al., Identification of prognostic alternative splicing signature in triple-negative breast cancer (2020).

[17] M. Shibata, M. Kanda, H. Tanaka, S. Umeda, T. Miwa, D. Shimizu, M. Hayashi, T. Inaishi, N. Miyajima, Y. Adachi, et al., Overexpression of derlin 3 is associated with malignant phenotype of breast cancer cells, Oncology reports 38 (2017) 1760–1766.

[18] L. J. Eichner, M.-C. Perry, C. R. Dufour, N. Bertos, M. Park, J. St-Pierre, V. Giguère, mir-378 mediates metabolic shift in breast cancer cells via the pgc-1β/errγ transcriptional pathway, Cell metabolism 12 (2010) 352–361.

[19] A. Dib, J. Adelaide, F. Courjal, A. Courseaux, J. Jacquemier, P. Gaudray, C. Theillet, M. Pebusque, D. Birnbaum, Coamplification in human breast-tumors and physical linkage at chromosomal band 12p13, of ccnd2 and fgf6 genes, International journal of oncology 5 (1994) 1375–1378.

[20] Y. Zhu, L. Yang, Q.-Y. Chong, H. Yan, W. Zhang, W. Qian, S. Tan, Z. Wu, P. E. Lobie, T. Zhu, Long noncoding rna linc00460 promotes breast cancer progression by regulating the mir-489-5p/fgf7/akt axis, Cancer Management and Research 11 (2019) 5983.

[21] A. Lyakhovich, N. Aksenov, P. Pennanen, S. Miettinen, M. H. Ahonen, H. Syvälä, T. Ylikomi, P. Tuohimaa, Vitamin d induced up-regulation of keratinocyte growth factor (fgf-7/kgf) in mcf-7 human breast cancer cells, Biochemical and Biophysical Research Communications 273 (2000) 675–680.

[22] J. Jacquemier, Z.-z. Sun, F. Penault-llorca, J. Geneix, E. Devilard, J. Adélaïde, D. Birnbaum, Fgf7 protein expression in human breast carcinomas, The Journal of Pathology: A Journal of the Pathological Society of Great Britain and Ireland 186 (1998) 269–274.

[23] H. Zhang, F. Meng, G. Liu, B. Zhang, J. Zhu, F. Wu, S. P. Ethier, F. Miller, G. Wu, Forkhead transcription factor foxq1 promotes epithelial–mesenchymal transition and breast cancer metastasis, Cancer research 71 (2011) 1292–1301.

[24] F. Meng, C. L. Speyer, B. Zhang, Y. Zhao, W. Chen, D. H. Gorski, F. R. Miller, G. Wu, Pdgfrα and β play critical roles in mediating foxq1-driven breast cancer stemness and chemoresistance, Cancer research 75 (2015) 584–593.

[25] X. Wu, G. Gardashova, L. Lan, S. Han, C. Zhong, R. T. Marquez, L. Wei, S. Wood, S. Roy, R. Gowthaman, et al., Targeting the interaction between rna-binding protein hur and foxq1 suppresses breast cancer invasion and metastasis, Communications biology 3 (2020) 1–16.

[26] F. A. Elian, U. Are, S. Ghosh, P. Nuin, T. Footz, T. P. McMullen, D. N. Brindley, M. A. Walter, Foxq1 is differentially expressed across breast cancer subtypes with low expression associated with poor overall survival, Breast cancer: targets and therapy 13 (2021) 171.

[27] G. Liliana, C. A. Suárez, D. Soto, A. Schiappacasse, D. Sapochnik, P. A. Sacca, G. Piwien Pilipuk, B. Peral, J. C. Calvo, Gal3st2 from mammary gland epithelial cells affects differentiation of 3t3-l1 preadipocytes (2014).

[28] M. Reyngold, S. Turcan, D. Giri, K. Kannan, L. A. Walsh, A. Viale, M. Drobnjak, L. T. Vahdat, W. Lee, T. A. Chan, Remodeling of the methylation landscape in breast cancer metastasis, PloS one 9 (2014) e103896.

[29] J. Masjkur, C. Arps-Forker, S. W. Poser, P. Nikolakopoulou, L. Toutouna, R. Chenna, T. Chavakis, A. Chatzigeorgiou, L.-S. Chen, A. Dubrovs-

ka, et al., Hes3 is expressed in the adult pancreatic islet and regulates gene expression, cell growth, and insulin release, Journal of Biological Chemistry 289 (2014) 35503–35516.

[30] L. Toutouna, P. Nikolakopoulou, S. W. Poser, J. Masjkur, C. Arps-Forker, M. Troullinaki, S. Grossklaus, V. Bosak, U. Friedrich, T. Ziemssen, et al., Hes3 expression in the adult mouse brain is regulated during demyelination and remyelination, Brain Research 1642 (2016) 124–130.

[31] K. J. Meaburn, P. R. Gudla, S. Khan, S. J. Lockett, T. Misteli, Disease-specific gene repositioning in breast cancer, Journal of cell biology 187 (2009) 801–812.

[32] G. L. Palmisano, M. P. Pistillo, P. Fardin, P. Capanni, G. Nicolò, S. Salvi, B. Spina, G. Pasciucco, G. B. Ferrara, Analysis of hla-g expression in breast cancer tissues, Human immunology 63 (2002) 969–976.

[33] E. M. de Kruijf, A. Sajet, J. G. van Nes, R. Natanov, H. Putter, V. T. Smit, G. J. Liefers, P. J. van den Elsen, C. J. van de Velde, P. J. Kuppen, Hla-e and hla-g expression in classical hla class i-negative tumors is of prognostic value for clinical outcome of early breast cancer patients, The Journal of Immunology 185 (2010) 7452–7459.

[34] D.-D. Dong, S.-m. Yie, K. Li, F. Li, Y. Xu, G. Xu, L. Song, H. Yang, Importance of hla-g expression and tumor infiltrating lymphocytes in molecular subtypes of breast cancer, Human immunology 73 (2012) 998–1004.

[35] S. Jeong, S. Park, B.-W. Park, Y. Park, O.-J. Kwon, H.-S. Kim, Human leukocyte antigen-g (hla-g) polymorphism and expression in breast cancer patients, PLoS one 9 (2014) e98284.

[36] P. Smith, L. J. Nicholson, N. Syed, A. Payne, L. Hiller, O. Garrone, M. Occelli, M. Gasco, T. Crook, Epigenetic inactivation implies independent functions for insulin-like growth factor binding protein (igfbp)-related protein 1 and the related igfbpl1 in inhibiting breast cancer phenotypes, Clinical cancer research 13 (2007) 4061–4068.

[37] I. Cheng, K. L. Penney, D. O. Stram, L. Le Marchand, E. Giorgi, C. A. Haiman, L. N. Kolonel, M. Pike, J. Hirschhorn, B. E. Henderson, et al., Haplotype-based association studies of igfbp1 and igfbp3 with prostate and breast cancer risk: the multiethnic cohort, Cancer epidemiology and prevention biomarkers 15 (2006) 1993–1997.

[38] A. H. Rosendahl, M. Hietala, M. Henningson, H. Olsson, H. Jernström, Igfbp1 and igfbp3 polymorphisms predict circulating igfbp-3 levels among women from high-risk breast cancer families, Breast cancer research and treatment 127 (2011) 785–794.

[39] J. H. Taube, N. Sphyris, K. S. Johnson, K. N. Reisenauer, T. A. Nesbit, R. Joseph, G. V. Vijay, T. R. Sarkar, N. A. Bhangre, J. J. Song, et al., The h3k27me3-demethylase kdm6a is suppressed in breast cancer stem-like cells, and enables the resolution of bivalency during the mesenchymal-epithelial transition, Oncotarget 8 (2017) 65548.

[40] H. Lu, Y. Xie, L. Tran, J. Lan, Y. Yang, N. L. Murugan, R. Wang, Y. J. Wang, G. L. Semenza, et al., Chemotherapy-induced s100a10 recruits kdm6a to facilitate oct4-mediated breast cancer stemness, The Journal of clinical investigation 130 (2020).

[41] G. Jeong, H. Bae, D. Jeong, J. Ham, S. Park, H. W. Kim, H.-S. Kang, S. J. Kim, A kelch domain-containing klhdc7b and a long non-coding rna st8sia6-as1 act oppositely on breast cancer cell proliferation via the interferon signaling pathway, Scientific reports 8 (2018) 1–10.

[42] P. Zhong, R. Shu, H. Wu, Z. Liu, X. Shen, Y. Hu, Low krt15 expression is associated with poor prognosis in patients with breast invasive carcinoma, Experimental and therapeutic medicine 21 (2021) 1–1.

[43] Y. Fang, L. Wang, C. Wan, Y. Sun, K. Van der Jeught, Z. Zhou, T. Dong, K. M. So, T. Yu, Y. Li, et al., Mal2 drives immune evasion in breast cancer by suppressing tumor antigen presentation, The Journal of Clinical Investigation 131 (2021).

[44] D. Dersh, J. W. Yewdell, et al., Immune mal2-practice: breast cancer immunoevasion via mhc class i degradation, The Journal of Clinical Investigation 131 (2021).

[45] J. Luo, S. Zeng, C. Tian, Morc4 promotes chemoresistance of luminal a/b breast cancer via stat3-mediated mid2 upregulation, OncoTargets and Therapy 13 (2020) 6795.

[46] R. Zeisig, T. Koklič, B. Wiesner, I. Fichtner, M. Sentjurč, Increase in fluidity in the membrane of mt3 breast cancer cells correlates with enhanced cell adhesion in vitro and increased lung metastasis in nod/scid mice, Archives of biochemistry and biophysics 459 (2007) 98–106.

[47] E. Dejardin, G. Bonizzi, A. Bellahcene, V. Castronovo, M.-P. Merville, V. Bours, Highly-expressed p100/p52 (nfkb2) sequesters other nf-kappa b-related proteins in the cytoplasm of human breast cancer cells., Oncogene 11 (1995) 1835–1841.

[48] S. K. Yeo, R. French, F. Spada, R. Clarkson, Opposing roles of nfkb2 gene products p100 and p52 in the regulation of breast cancer stem cells, Breast cancer research and treatment 162 (2017) 465–477.

[49] F. Journe, V. Durbecq, C. Chaboteaux, G. Rouas, G. Laurent, D. Nonclercq, C. Sotiriou, J.-J. Body, D. Larsimont, Association between farnesoid x receptor expression and cell proliferation in estrogen receptor-positive luminal-like breast cancer from postmenopausal patients, Breast cancer research and treatment 115 (2009) 523–535.

[50] F. Journe, G. Laurent, C. Chaboteaux, D. Nonclercq, V. Durbecq, D. Larsimont, J.-J. Body, Farnesol, a mevalonate pathway intermediate, stimulates mcf-7 breast cancer cell growth through farnesoid-x-receptor-mediated estrogen receptor activation, Breast cancer research and treatment 107 (2008) 49–61.

[51] E. J. Mucaki, K. Baranova, H. Q. Pham, I. Rezaeian, D. Angelov, A. Ngom, L. Rueda, P. K. Rogan, Predicting outcomes of hormone and chemotherapy in the molecular taxonomy of breast cancer international consortium (metabric) study by biochemically-inspired machine learning, F1000Research 5 (2016).

[52] Y. Wu, D.-d. Yu, D.-l. Yan, Y. Hu, D. Chen, Y. Liu, H.-d. Zhang, S.-r. Yu, H.-x. Cao, J.-f. Feng, Liver x receptor as a drug target for the treatment of breast cancer, Anti-Cancer Drugs 27 (2016) 373–382.

[53] Y.-k. Song, Y. Wang, Y.-y. Wen, P. Zhao, Z.-j. Bian, Microrna-22 suppresses breast cancer cell growth and increases paclitaxel sensitivity by targeting nras, Technology in cancer research & treatment 17 (2018) 1533033818809997.

[54] D. Cimino, C. De Pitta, F. Orso, M. Zampini, S. Casara, E. Penna, E. Quaglino, M. Forni, C. Damasco, E. Pinatel, et al., mir148b is a major coordinator of breast cancer progression in a relapse-associated microrna signature by targeting itga5, rock1, pik3ca, nras, and csf1, The FASEB Journal 27 (2013) 1223–1235.

[55] B. Sharif-Askari, L. Amrein, R. Aloyz, L. Panasci, Parp3 inhibitors me0328 and olaparib potentiate vinorelbine sensitization in breast cancer cell lines, Breast cancer research and treatment 172 (2018) 23–32.

[56] L.-M. Dong, X.-L. Zhang, M.-H. Mao, Y.-P. Li, X.-Y. Zhang, D.-W. Xue, Y.-L. Liu, Linc00511/mirna-143-3p modulates apoptosis and malignant phenotype of bladder carcinoma cells via pcmt1, Frontiers in cell and developmental biology 9 (2021).

[57] D. Fu, C. He, J. Wei, Z. Zhang, Y. Luo, H. Tan, C. Ren, Pgk1 is a potential survival biomarker and invasion promoter by regulating the

hif-1α–mediated epithelial-mesenchymal transition process in breast cancer, Cellular physiology and biochemistry 51 (2018) 2434–2444.

[58] Y. He, Y. Luo, D. Zhang, X. Wang, P. Zhang, H. Li, S. Ejaz, S. Liang, Pgk1-mediated cancer progression and drug resistance, American Journal of Cancer Research 9 (2019) 2280.

[59] S. Sun, H. Wu, X. Wu, Z. You, Y. Jiang, X. Liang, Z. Chen, Y. Zhang, W. Wei, Y. Jiang, et al., Silencing of pgk1 promotes sensitivity to paclitaxel treatment by upregulating xaf1-mediated apoptosis in triple-negative breast cancer, Frontiers in Oncology 11 (2021) 539.

[60] Y. Bao, L. Wang, L. Shi, F. Yun, X. Liu, Y. Chen, C. Chen, Y. Ren, Y. Jia, Transcriptome profiling revealed multiple genes and ecm-receptor interaction pathways that may be associated with breast cancer, Cellular & molecular biology letters 24 (2019) 1–20.

[61] J. Li, K. Hu, D. He, L. Zhou, Z. Wang, Y. Tao, Prognostic value of plxnd1 and tgf-β1 coexpression and its correlation with immune infiltrates in hepatocellular carcinoma, Frontiers in Oncology 10 (2021) 2972.

[62] R. Medina-Aguilar, C. Pérez-Plasencia, P. Gariglio, L. A. Marchat, A. Flores-Pérez, C. López-Camarillo, J. G. Mena, Dna methylation data for identification of epigenetic targets of resveratrol in triple negative breast cancer cells, Data in Brief 11 (2017) 169–182.

[63] Y. Gu, G. Chen, Y. Du, Screening of prognosis-related genes in primary breast carcinoma using genomic expression data, Journal of Computational Biology 27 (2020) 1030–1040.

[64] L.-X. Yan, X.-F. Huang, Q. Shao, M.-Y. Huang, L. Deng, Q.-L. Wu, Y.-X. Zeng, J.-Y. Shao, Microrna mir-21 overexpression in human breast cancer is associated with advanced clinical stage, lymph node metastasis and patient poor prognosis, RNA 14 (2008) 2348–2360.

[65] D. Bem, S.-I. Yoshimura, R. Nunes-Bastos, F. F. Bond, M. A. Kurian, F. Rahman, M. T. Handley, Y. Hadzhiev, I. Masood, A. A. Straatman-Iwanowska, et al., Loss-of-function mutations in rab18 cause warburg micro syndrome, The American journal of human genetics 88 (2011) 499–507.

[66] X. Chen, J. Xu, F. Zeng, C. Yang, W. Sun, T. Yu, H. Zhang, Y. Li, Inferring cell subtypes and lncrna function by a cell-specific cerna network in breast cancer, Frontiers in oncology 11 (2021).

[67] J. Jiang, W. Chang, Y. Fu, Y. Gao, C. Zhao, X. Zhang, S. Zhang, Sav1, regulated by microrna-21, suppresses tumor growth in colorectal cancer, Biochemistry and Cell Biology 97 (2019) 91–99.

[68] H. J. Chan, H. Li, Z. Liu, Y.-C. Yuan, J. Mortimer, S. Chen, Serpina1 is a direct estrogen receptor target gene and a predictor of survival in breast cancer patients, Oncotarget 6 (2015) 25815.

[69] S. Mandal, N. Gamit, L. Varier, A. Dharmarajan, S. Warrier, Inhibition of breast cancer stem-like cells by a triterpenoid, ursolic acid, via activation of wnt antagonist, sfrp4 and suppression of mirna-499a-5p, Life sciences 265 (2021) 118854.

[70] C. L. Enriori, W. Orsini, M. del Carmen Cremona, A. E. Etkin, L. R. Cardillo, J. Reforzo-Membrives, Decrease of circulating level of shbg in postmenopausal obese women as a risk factor in breast cancer: reversible effect of weight loss, Gynecologic oncology 23 (1986) 77–86.

[71] C.-A. Wang, D. Drasin, C. Pham, P. Jedlicka, V. Zaberezhnyy, M. Guney, H. Li, R. Nemenoff, J. C. Costello, A.-C. Tan, et al., Homeoprotein six2 promotes breast cancer metastasis via transcriptional and epigenetic control of e-cadherin expression, Cancer research 74 (2014) 7357–7370.

[72] Q. Jia, L. Ye, S. Xu, H. Xiao, S. Xu, Z. Shi, J. Li, Z. Chen, Circular rna 0007255 regulates the progression of breast cancer through mir-335-5p/six2 axis, Thoracic cancer 11 (2020) 619–630.

[73] I. J. Lafta, STAG3 gene expression in breast cancer cells, Ph.D. thesis, University of Sheffield, 2016.

[74] I. J. Lafta, H. E. Bryant, A. S. Goldman, Investigating stag3 expression as a novel human breast cancer biomarker (????).

[75] Y. Lin, C. Cui, M. Su, L. K. Silbart, H. Liu, J. Zhao, L. He, Y. Huang, D. Xu, X. Wei, et al., Identification of tapbpl as a novel negative regulator of t-cell function, EMBO molecular medicine 13 (2021) e13404.

[76] M. Cappetta, L. Fernandez, L. Brignoni, N. Artagaveytia, C. Bonilla, M. López, M. Esteller, B. Bertoni, M. Berdasco, Discovery of novel dna methylation biomarkers for non-invasive sporadic breast cancer detection in the latino population, Molecular oncology 15 (2021) 473–486.

[77] E. H. Gort, K. P. Suijkerbuijk, S. M. Roothaan, V. Raman, M. Vooijs, E. Van Der Wall, P. J. Van Diest, Methylation of the twist1 promoter, twist1 mrna levels, and immunohistochemical expression of twist1 in breast cancer, Cancer Epidemiology and Prevention Biomarkers 17 (2008) 3325–3330.

[78] Q.-Q. Li, J.-D. Xu, W.-J. Wang, X.-X. Cao, Q. Chen, F. Tang, Z.-Q. Chen, X.-P. Liu, Z.-D. Xu, Twist1-mediated adriamycin-induced epithelial-mesenchymal transition relates to multidrug resistance and invasive potential in breast cancer cells, Clinical cancer research 15 (2009) 2657–2665.

[79] L. Ai, W.-J. Kim, M. Alpay, M. Tang, C. E. Pardo, S. Hatakeyama, W. S. May, M. P. Kladde, C. D. Heldermon, E. M. Siegel, et al., Trim29 suppresses twist1 and invasive breast cancer behavior, Cancer research 74 (2014) 4875–4887.

[80] Y. Xu, L. Qin, T. Sun, H. Wu, T. He, Z. Yang, Q. Mo, L. Liao, J. Xu, Twist1 promotes breast cancer invasion and metastasis by silencing foxa1 expression, Oncogene 36 (2017) 1157–1166.

[81] D. Agarwal, S. Goodison, B. Nicholson, D. Tarin, V. Urquidi, Expression of matrix metalloproteinase 8 (mmp-8) and tyrosinase-related protein-1 (tyrp-1) correlates with the absence of metastasis in an isogenic human breast cancer model, Differentiation 71 (2003) 114–125.

[82] R. A. Van de Ven, M. Tenhagen, W. Meuleman, J. J. van Riel, R. C. Schackmann, P. W. Derksen, Nuclear p120-catenin regulates the anoikis resistance of mouse lobular breast cancer cells through kaiso-dependent wnt11 expression, Disease models & mechanisms 8 (2015) 373–384.

[83] K. Menck, S. Heinrichs, D. Wlochowitz, M. Sitte, H. Noeding, A. Janshoff, H. Treiber, T. Ruhwedel, B. Schatlo, C. von der Brelie, et al., Wnt11 is a novel ligand for ror2 in human breast cancer, bioRxiv (2020).

[84] S. Maubant, B. Tesson, V. Maire, M. Ye, G. Rigaill, D. Gentien, F. Cruzalegui, G. C. Tucker, S. Roman-Roman, T. Dubois, Transcriptome analysis of wnt3a-treated triple-negative breast cancer cells, PLoS One 10 (2015) e0122333.

[85] V. Eterno, A. Zambelli, L. Villani, A. Tuscano, S. Manera, A. Spitaleri, L. Pavesi, A. Amato, Aurka controls self-renewal of breast cancer-initiating cells promoting wnt3a stabilization through suppression of mir-128, Scientific Reports 6 (2016) 1–13.

[86] R. I. Aqeilan, V. Donati, E. Gaudio, M. S. Nicoloso, M. Sundvall, A. Korhonen, J. Lundin, J. Isola, M. Sudol, H. Joensuu, et al., Association of wwox with erbb4 in breast cancer, Cancer Research 67 (2007) 9330–9336.

[87] E. Płuciennik, R. Kusińska, P. Potemski, R. Kubiak, R. Kordek, A. Bednarek, Wwoxthe fra16d cancer gene: expression correlation with breast cancer progression and prognosis, European Journal of Surgical Oncology (EJSO) 32 (2006) 153–157.

[88] M. I. Nunez, J. Ludes-Meyers, M. C. Abba, H. Kil, N. W. Abbey, R. E. Page, A. Sahin, A. J. Klein-Szanto, C. M. Aldaz, Frequent loss of wwox expression in breast cancer: correlation with estrogen receptor status, Breast cancer research and treatment 89 (2005) 99–105.
