# Supplementary figures and images for: Detecting prognostic biomarkers of breast cancer by regularized Cox proportional hazards models
Source: J Transl Med. 2021 Dec 20;19:514. doi: 10.1186/s12967-021-03180-y (PMC8686664; doi:10.1186/s12967-021-03180-y)

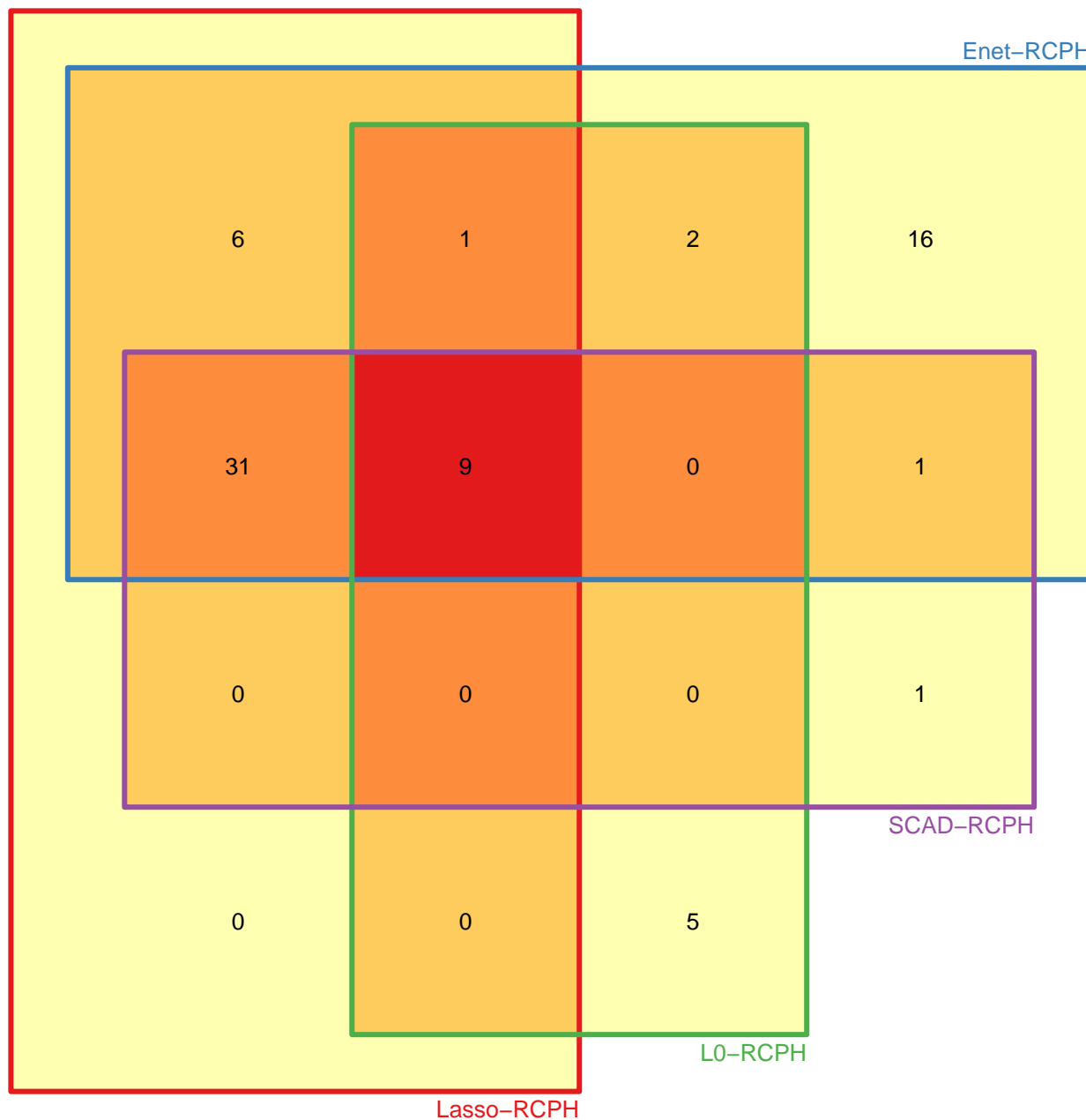

Supplement: Supplementary file 6 — Additional file 6: Figure S6. The overlap genes in the four feature subsets identified by Lasso-RCPH, Enet-RCPH, L0-RCPH and SCAD-RCPH. [file 12967_2021_3180_MOESM6_ESM.pdf]
